# Bacterial Carriers for Glioblastoma Therapy

**DOI:** 10.1016/j.omto.2016.12.003

**Published:** 2016-12-14

**Authors:** Nalini Mehta, Johnathan G. Lyon, Ketki Patil, Nassir Mokarram, Christine Kim, Ravi V. Bellamkonda

**Affiliations:** 1 Wallace H. Coulter Department of Biomedical Engineering, Georgia Institute of Technology and Emory School of Medicine, UA Whitaker Building, 313 Ferst Drive, Atlanta, GA 30332, USA; 2 Department of Biomedical Engineering, Pratt School of Engineering, Duke University, 101 Science Drive, Durham, NC 27708-0271, USA

**Keywords:** brain cancer, bacteria, drug delivery, tumor targeting, glioblastoma, *Salmonella typhimurium*, p53, Azurin, VNP 20009, RNU rats

## Abstract

Treatment of aggressive glioblastoma brain tumors is challenging, largely due to diffusion barriers preventing efficient drug dosing to tumors. To overcome these barriers, bacterial carriers that are actively motile and programmed to migrate and localize to tumor zones were designed. These carriers can induce apoptosis via hypoxia-controlled expression of a tumor suppressor protein p53 and a pro-apoptotic drug, Azurin. In a xenograft model of human glioblastoma in rats, bacterial carrier therapy conferred a significant survival benefit with 19% overall long-term survival of >100 days in treated animals relative to a median survival of 26 days in control untreated animals. Histological and proteomic analyses were performed to elucidate the safety and efficacy of these carriers, showing an absence of systemic toxicity and a restored neural environment in treated responders. In the treated non-responders, proteomic analysis revealed competing mechanisms of pro-apoptotic and drug-resistant activity. This bacterial carrier opens a versatile avenue to overcome diffusion barriers in glioblastoma by virtue of its active motility in extracellular space and can lead to tailored therapies via tumor-specific expression of tumoricidal proteins.

## Introduction

The diffuse and infiltrative nature of glioblastoma (GBM) tumors presents unique challenges for effective treatment. Although surgical resection, radiation, and systemic chemotherapy are the clinical standards of care, the median survival of patients is less than 15 months primarily due to tumor recurrence.^1^ Systemic delivery of chemotherapeutics is not effective due to the diffusion barriers imposed by the solid tumor,^2^, ^3^ which results in a suboptimal therapeutic concentration of the cancer drug within the tumor. The dominant strategy to overcome diffusion barriers is convection enhanced delivery (CED) whereby a pressure gradient enhances interstitial infusion of drugs.^4^ Not only is it challenging to accurately model drug dosing with this method given brain tissue heterogeneity,^5^, ^6^ but also the increased pressure within the brain caused by CED could lead to cerebral edema, hemiparesis, and other neurological damage.^7^ Therefore, there is a critical need to explore alternate approaches to overcome diffusion barriers that impede adequate drug dosing into solid tumors.

Engineered nanoparticles have been shown to selectively detect and destroy tumor cells.^8^, ^9^, ^10^ Although promising, nanoparticle therapy is still limited by physiological obstacles such as protein adsorption, phagocytic sequestration, renal clearance, as well as physical obstacles such as diffusion, flow, and shear forces, which limit diffusion of nanoparticles to or within tumors.^11^, ^12^, ^13^, ^14^, ^15^ In fact, a comprehensive analysis of data from 10 years has indicated that only 0.7% (median) of the administered dose of nanoparticles reach solid tumors.^16^ In this study, we report the design of a bacterial carrier, an avirulent strain of Salmonella *typhimurium*, with active motility in extracellular space and within brain tumors, and the capability of expressing apoptotic proteins within the tumor.

The bacterial carrier has mutation in the *msbB* gene that is required for terminal myristoylation of lipid A, a component of the outer membrane in Gram-negative bacteria such as enteropathogenic *Escherichia coli* and *S. typhimurium*.^17^ Lipid A is necessary for tumor necrosis factor alpha (TNF-α)-mediated septic shock during food poisoning caused by these bacteria.^18^, ^19^ A mutation in *msbB* gene impedes systemic toxicity as evident from in vivo evaluation of this strain in mice.^20^, ^21^ This strain has also been used in clinical trials in patients with metastatic melanoma.^22^ However, in these clinical trials, no antitumor effect was observed at a maximum patient-tolerated dose of 3 × 10^8^ cfu/m^2^ given intravenously. Therefore, although safe, the mere presence of the bacterial strain is insufficient to elicit a tumoricidal response.

Tumor-specific localization of this strain is enabled by a mutation in the *purI* gene (encodes for purine biosynthetic enzyme), which makes the strain deficient in synthesizing purines, creating a need for external sources of purines for its survival and multiplication.^23^, ^24^ Tumors are a rich source of purines with adenosine triphosphate concentrations in the range of hundreds of micromolar, whereas healthy tissue concentrations are below detectable levels.^25^, ^26^, ^27^ Thus, given the combination of purine deficiency in the carriers and relative purine abundance in tumors, the carrier exhibits a tumor-seeking propensity.

The bacterial carrier was designed to express wild-type tumor suppressor protein p53, a multifaceted protein that exerts its apoptotic activity through a cooperative effect of transcription-dependent and transcription-independent pathways.^28^, ^29^, ^30^, ^31^, ^32^ In the transcription-dependent pathway, phosphorylated p53 transcriptionally up-regulates a number of pathways that express pro-apoptotic proteins such as BAX, PUMA, NOX, and suppresses anti-apoptotic proteins such as BCL2 and BCLX_L_. The pro-apoptotic proteins induce permeabilization of outer mitochondrial membrane (OMM) enabling the release of cytochrome *c* (CYCS) and other apoptotic proteins leading to apoptosis. In the transcription-independent pathway, p53 directly activates BAX, which undergoes a conformational switch, oligomerizes, and inserts into OMM leading to apoptosis.^33^, ^34^ The significance of p53 in cancer is evident from the fact that more than 50% of cancers with poor clinical prognosis are associated with mutations in the p53 core DNA-binding domain.^35^, ^36^, ^37^ In patients with primary and secondary GBM, the incidence of p53 mutations is 28% and 65%, respectively.^38^, ^39^ Several treatment strategies have tried to reactivate the mutated p53, to reconstitute wild-type p53, or to inhibit MDM2 (protein that down-regulates p53).^40^, ^41^, ^42^ However, absence of an efficient delivery system into solid tumors has dwarfed the effectiveness of these therapies.

To enhance the apoptotic activity of the carrier as well as to increase the stability of wild-type p53, bacterial carrier that additionally expresses Azurin was designed. Azurin is a multifunctional protein that induces apoptosis of cancerous cells, inhibits receptor tyrosine kinase-mediated cell signaling and angiogenesis, and is a promising tumoricidal protein.^43^, ^44^, ^45^ Azurin stabilizes p53 by forming a complex and protecting it from undergoing ubiquitination and proteasomal degradation.^46^, ^47^ This is important, as the intracellular half-life of p53 is approximately 25–30 min if left unprotected.^46^, ^47^, ^48^ Although full-length Azurin is a potent tumoricidal protein, it does not penetrate GBM cells, nor does it cross the blood-brain barrier in mice when injected intraperitoneally with the pure protein.^49^, ^50^ Additionally Azurin is not cytotoxic to non-cancerous healthy cells. A small synthetic peptide (p28) derived from full-length Azurin sequence has been used in phase I clinical trials;^51^ however, the p28 peptide does not possess the inhibitory potential of full-length Azurin because there are other non-overlapping domains (p26) within the protein that confer anti-cancer activity specifically targeting tyrosine kinases.^43^, ^50^

**Figure 1:**
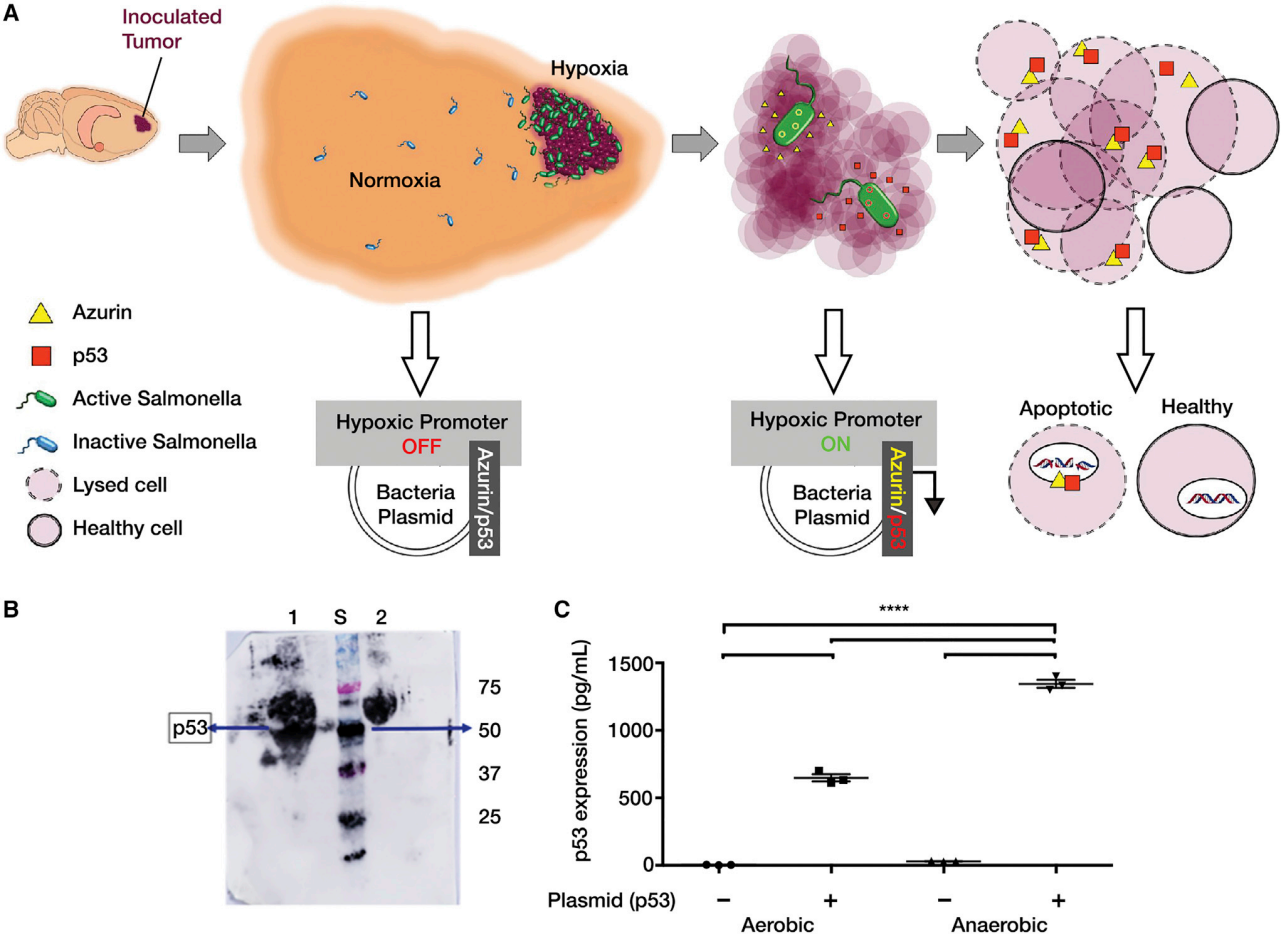
Bacterial Carrier Design (A) Schematic showing hypoxia-inducible expression of p53 and Azurin within the tumor by carrier where conditions are hypoxic compared to the surrounding healthy tissues, thus causing selective apoptosis within tumor cells. (B) Western blotting showed relatively greater expression of p53 (around 50 kDa) in vitro when the carrier was grown anaerobically (lane 1) compared to when grown aerobically (lane 2). STDs (S) used: precision plus protein dual-color standards from Bio-Rad. (C) ELISA showing relatively greater expression of p53 when carrier was grown anaerobically compared to when grown aerobically in vitro. Control used is carrier without the expression plasmid. Mean ± SEM shown.

Finally, to constrain expression of these tumoricidal proteins to the tumor region, both p53 and Azurin expression by the carriers was engineered to be driven by a hypoxic promoter *pflE*.^52^ This approach exploits the hypoxic tumor microenvironment combined with purine-dependent motile carrier to achieve tumor-targeted expression of p53 and full-length Azurin to induce apoptosis of glioblastoma cells (Figure 1A).

## Results

### Expression of Azurin and p53 from Hypoxia-Inducible *pflE* Promoter Occurs Abundantly under Anaerobic Conditions

The postulated mechanism by which p53- and Azurin-expressing carriers induce apoptosis within tumors is depicted in Figure 1A. Restricted expression of apoptotic proteins Azurin and p53 by hypoxia-inducible *pflE* promoter in *S. typhimurium* strain VNP 20009 (carrier) was attained using specific primers (Table S1). Prior to animal studies, in vitro expression of p53 was confirmed using western blotting after growing the carrier under aerobic and anaerobic conditions (BD Anaerobic GasPak EZ system) (Figure 1B). p53 expression was also confirmed by an ELISA (Figure 1C). *pflE* promoter is not completely shut down under aerobic conditions; therefore, minimal p53 expression was observed under aerobic conditions.^52^, ^53^ The expression of Azurin was confirmed using mass spectrometry (Table S2).

### Injection of Carriers with Combined Action of Azurin and p53 Suppressed Tumor Growth and Led to Increased Survival

**Figure 2:**
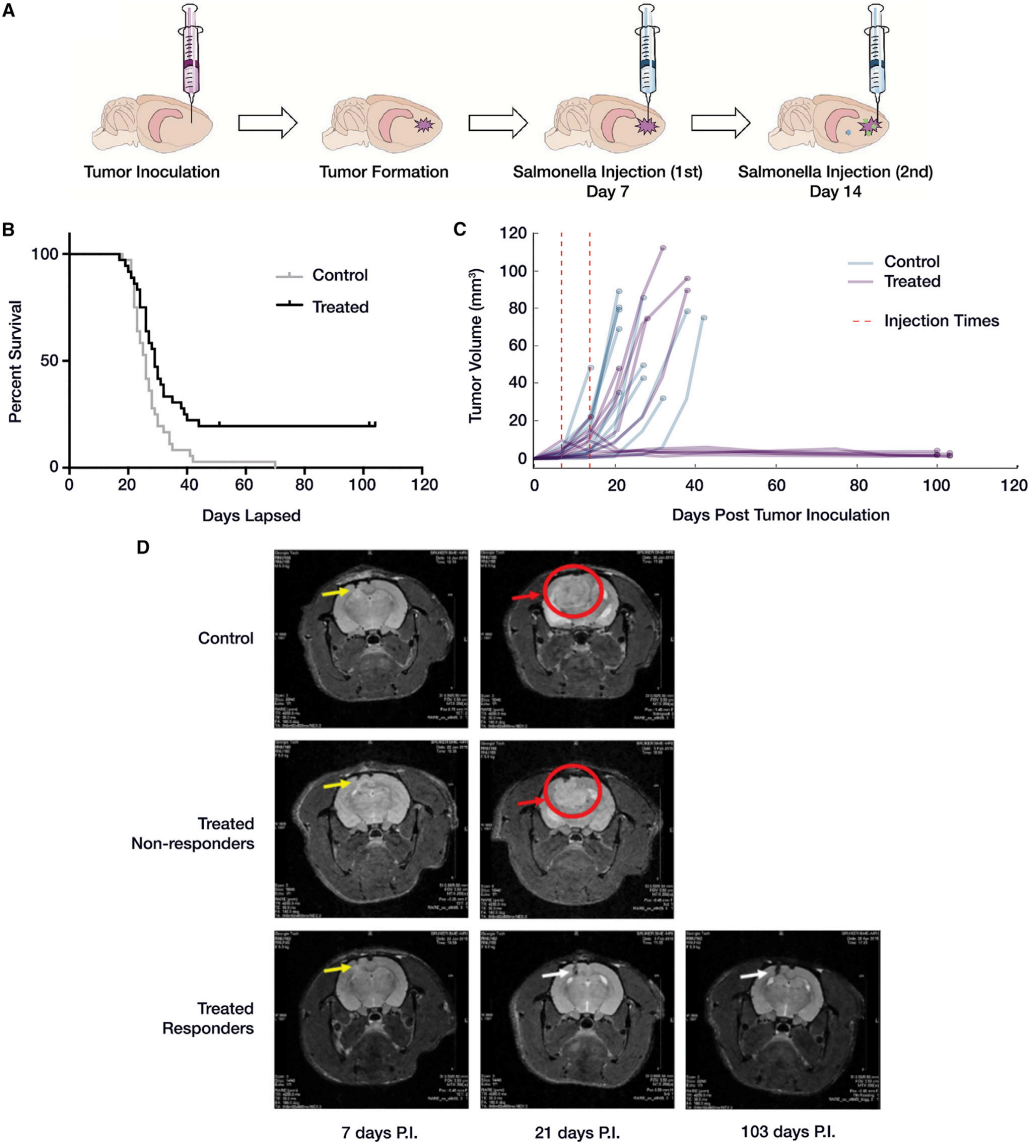
In Vivo Outcomes of Bacterial Carrier Therapy (A) Schematic showing timeline of intracranial tumor implantation followed by injection of carrier at the same site of tumor implant after 1 week. Two injections of carrier were given a week apart from one another. (B) Compiled Kaplan-Meier plot for control (sham injected, n = 36) and treated rats (injected with carrier expressing Azurin plus p53, n = 36). Results showed around 19.4% survival in treated rat group (responders) with survival extending beyond 100 days. Log rank Mantel-Cox test p value = 0.0046. (C) Tumor volumes calculated from MRI images for two sets of experiments involving control (n = 12) and treated (n = 12) rats. Tumor volume increased exponentially in all the control rats. In 6 out of 12 treated rats (responders), the tumor growth was suppressed, which led to increased survival. (D) Representative MRI images of brain from control, treated non-responder, and responder rats. Yellow arrows show tumor implant site, which is the same site for carrier injection. In the control and treated non-responder rats, tumor continued to grow as shown here on day 21 post-tumor implant (PI) (red arrow). However, in some of the treated rats, the tumor growth was suppressed (responder), and only slight inflammation was observed at needle site on day 103 PI (white arrow).

The timeline of carrier injection following tumor implantation is shown in Figure 2A. Carriers capable of expressing both p53 and Azurin were injected intracranially at the tumor implant site to a depth of 1.5 mm using a stereotax on days 7 and 14 post-tumor implant. Overall survival was 19% in treated rats, and 0% in control rats (Figure 2B). Compiled Kaplan-Meier curves over six different animal experiments showed significant difference in survival using a log-rank Mantel-Cox test (p < 0.0046), although only a 3-day difference was observed in median survival between control (26 days, n = 36) and treated (29 days, n = 36) rats.

Tumor volume was assessed by MRI for 12 control and 12 treated rats from two distinct experimental cohorts that had sufficient number of responders for further analysis. An exponential increase in tumor volume was observed for all 12 control rats, whereas 6 (responders) out of 12 treated rats demonstrated tumor suppression (Figure 2C) corresponding with overall survival. MRI brain scans of treated responder rats that were injected in the left cortex with *both* Azurin- and p53-expressing carrier showed dramatic tumor regression following carrier injection compared to control and treated non-responder rats (Figures 2B and 2D). No tumor was readily discriminable by MRI measurement for treated responder rats after 40 days. Another set of studies with rats that were treated singly with carrier expressing either p53 or Azurin (but not both) showed increased tumor growth with no survival (Figures S1A–S1D).

### Fluorescent in Situ Hybridization Demonstrates Carrier Migration and Colonization into Deep Tumors

To follow the migration of the intracranially injected carrier, a *Salmonella-*specific, fluorescently labeled 23S rDNA probe was used (Table S1).

**Figure 3:**
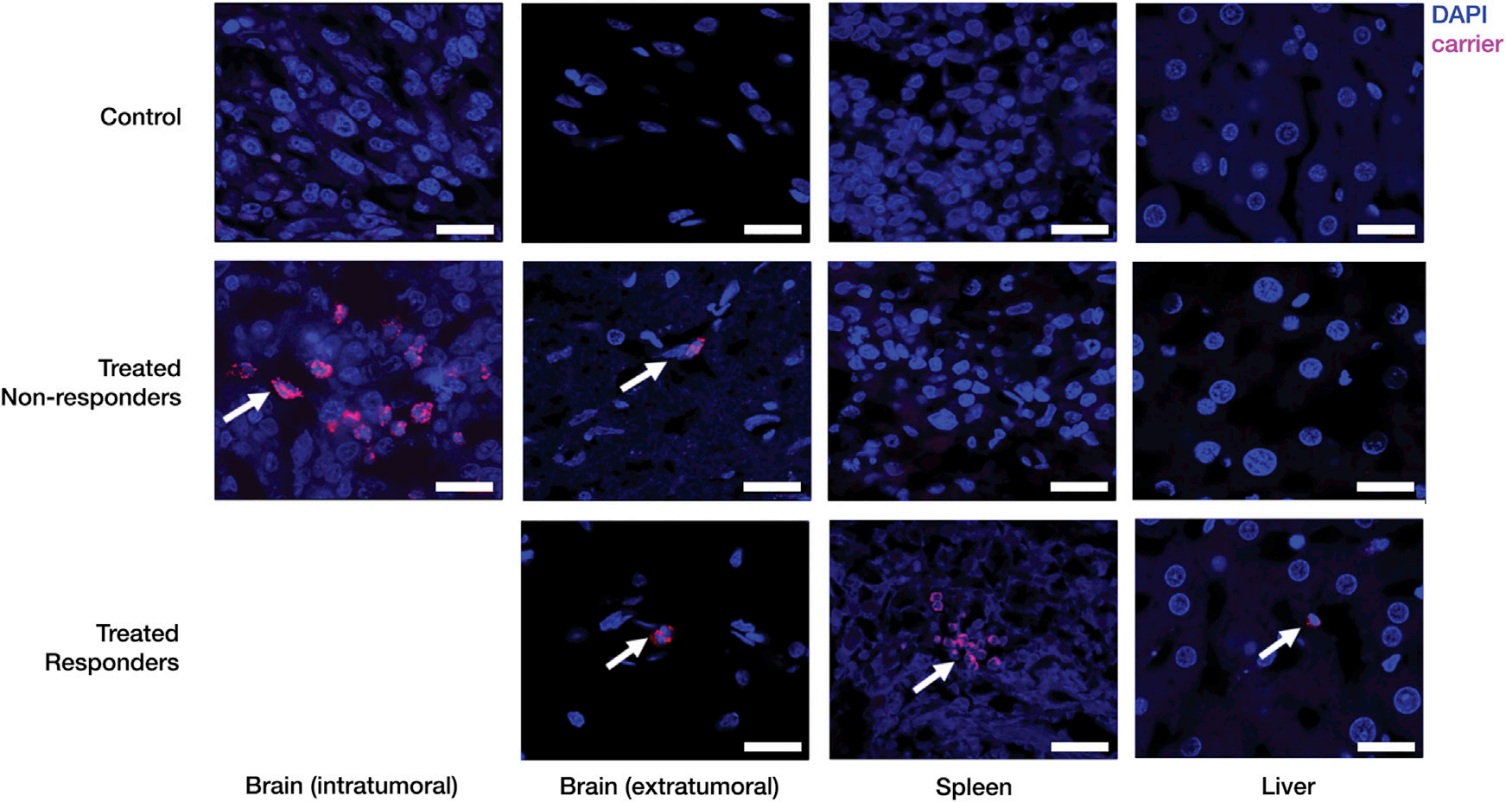
Biodistribution of Bacterial Carriers Fluorescent in situ hybridization (FISH) to detect carrier (pink) in the brain, spleen, and liver of treated non-responder and treated responder rats (the latter, euthanized on day 104). A number of carrier cells were present *inside the tumor* of treated non-responder rats (white arrows), with negligible number in the surrounding healthy tissue. Carriers were not observed in the spleen and liver of treated non-responders. For responders, significantly few carriers were present in the brain and liver. Carriers were detected in the spleens of responders (white arrows). Scale bars, 20 μm.

In the treated responder rats where the tumor growth was suppressed, negligible number of carriers were observed within the brain and the liver after 100 days post-tumor implant. However, a few carriers were observed in the spleen, indicating that, in the absence of a tumor, carriers have a greater tendency to relocate to the spleen (Figure 3). Additionally, the treated responder rats did not show any sign of illness even though the carrier was present in the spleen likely due to the *msbB* mutation that limits its ability to elicit systemic toxicity.^23^

In the treated non-responder rat brain, a number of carriers were detected within the tumor region, with minimal detection in the surrounding healthy tissue. This indicates that bacterial carriers could easily migrate throughout the tumor and preferentially colonize it compared to the surrounding tissue. The spleen and liver of treated non-responder rats did not show presence of the carrier (Figure 3).

### VNP 20009 Strains Were Recovered from Brains of Immunocompetent Rats

The colony counts obtained from rat brains (n = 3, without tumor inoculation) on day 15 after bacterial injection were at least 10-fold higher relative to the number of bacteria inoculated. This indicates that the strain survives even when challenged by host immune response.

### Carrier Injection Did Not Elevate TNF-α Levels in Blood, Brain, Spleen, and Liver of Treated Rats, Indicating Absence of Systemic Toxicity; Apoptosis Was Not Observed in Liver and Spleen

**Figure 4:**
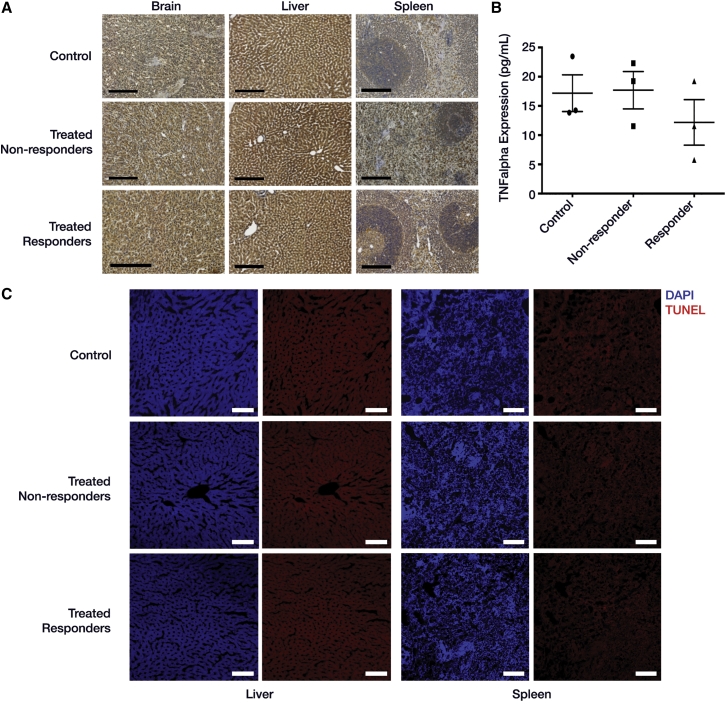
Bacterial Carriers Exhibit Lack of Systemic Toxicity (A) DAB staining with anti-TNF-α (indicator of systemic toxicity) for brain, liver, and spleen sections from control, treated non-responder, and responder rats looked comparable. (B) TNF-α levels were comparable and not significantly different in serum from control, treated non-responder, and responder rats as observed in ELISA (n = 3 for each group), indicating no increase in systemic toxicity due to carrier treatment (ANOVA, alpha = 0.05, Tukey multiple comparisons; control versus non-responders: p = 0.9939; control versus responders: p = 0.5867; non-responders versus responders: p = 0.5291). Mean ± SEM and pre-averaged biological replicates shown. (C) TUNEL staining showed absence of apoptosis within spleen and liver of control, treated non-responder, and responder rats. DAPI (blue), TUNEL (red; however, what is shown above contains only background). Scale bars, 200 μm (black) and 100 μm (white).

Although strains of *S. typhimurium* have been used as anti-cancer agents for treating a variety of cancers,^54^ one potential concern is their ability to induce systemic toxicity via the endotoxic lipid A membrane component. To mitigate this risk, VNP20009 strain was chosen because it is deficient in the lipid A component.^23^ A 3,3′-diaminobenzidine (DAB) peroxidase staining was performed with anti-TNF-α antibodies on brain, liver, and spleen sections to check for levels of TNF-α as an indicator of systemic toxicity. No difference was observed between the three groups (Figure 4A). To further rule out any signs of systemic toxicity in carrier-injected rats, an ELISA was performed on the serum (collected post-euthanization) to check for levels of TNF-α. It was seen that TNF-α levels were comparable between control, treated responder and non-responder rats (Figure 4B). Terminal deoxynucleotidyl transferase dUTP nick end labeling (TUNEL) staining performed for liver and spleen of control, treated non-responder, and responder rats did not show any signs of apoptosis (Figure 4C).

### p53 Is Present in Non-responder Tumors and Coincides with Expression of Pro-apoptotic markers, but Also Proliferative Ones

**Figure 5:**
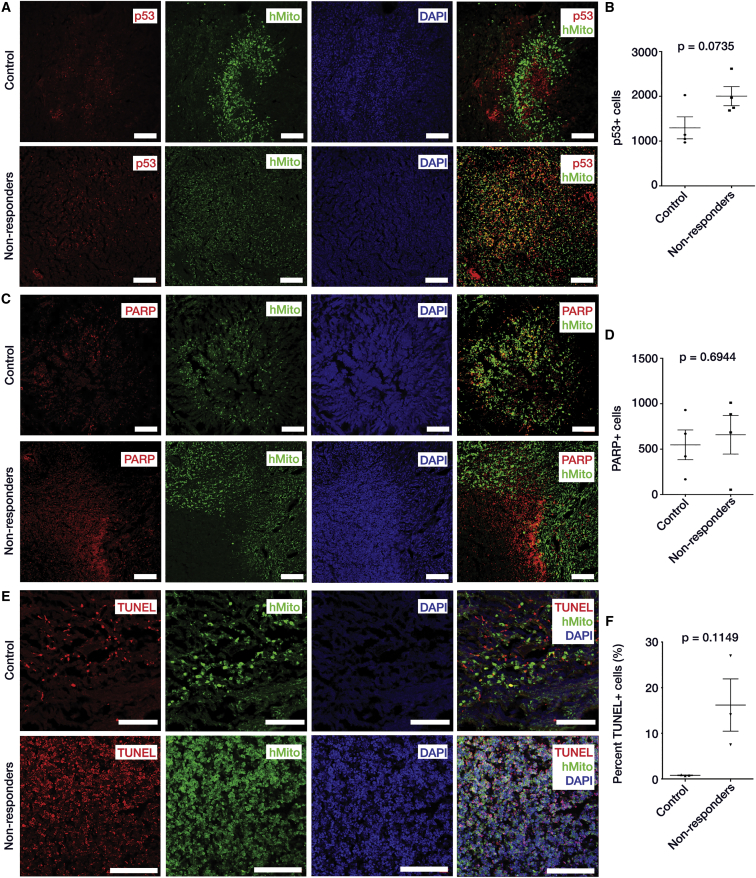
Immunohistochemistry of p53-Mediated Apoptosis after Bacterial Carrier Therapy (A) IHC images of brain sections from control and treated non-responder rats to check for expression of p53. Green, human anti-mitochondria antibodies that specifically stain tumor cells derived from U87MG human tumor cell line. Blue, DAPI; and red, p53. More p53 observed within tumor of treated non-responder rats compared to control. (B) Quantification of IHC images showed greater p53 levels in treated non-responder rats compared to control rats with p value of 0.0735. Nine images were taken from each control (n = 4) and treated non-responder rat (n = 4) for this analysis. (C) IHC images of brain sections from control and treated non-responder rats to check for presence of cleaved PARP (indicator of apoptosis). Green, human anti-mitochondria antibodies; blue, DAPI; and red, cleaved PARP. There were more PARP-positive cells in treated non-responder rats compared to control. (D) Quantification of IHC images showed more cleaved PARP in treated non-responder rats compared to controls with p value of 0.6944. Nine images were taken from each control (n = 4) and treated non-responder rat (n = 4) for this analysis. (E) TUNEL staining to check for apoptosis within tumor of control and treated non-responder rats. Green, anti-mitochondria antibodies; blue, DAPI; and red, TUNEL-positive cells. There were more TUNEL-positive cells in the tumor region of treated non-responders compared to control. (F) Quantification and statistical analysis of TUNEL taken from 18 different regions within each tumor of control (n = 3) and treated non-responder (n = 3) rats. TUNEL reaction in treated non-responders was 8- to 10-fold greater compared to control rats. All statistics were down by t test with Welch’s correction, alpha = 0.05. Mean ± SEM and pre-averaged biological replicates shown. Scale bars, 100 μm.

Fluorescent staining of brain sections using anti-p53 antibodies (Figure 5A) showed greater levels of p53 in treated non-responder rats compared to control rats, although the difference was not statistically significant (Figure 5B). Unfortunately, fluorescent staining for Azurin was not technically possible due to commercial unavailability of anti-Azurin antibodies.

To investigate whether expression of p53 and Azurin led to changes in the extent of apoptosis, immunohistochemistry (IHC) with anti-cleaved poly-ADP ribose polymerase (PARP) antibodies (indicator of apoptosis) and TUNEL assay were performed. Extent of PARP staining within tumors (Figure 5C) was slightly greater, but not significant in treated non-responder rats compared to the control rats (p < 0.6944) (Figure 5D). However, treated non-responder rats showed an 8- to 10-fold increase in the number of TUNEL-positive cells within the tumor region compared to the control rats (p < 0.1149) (Figures 5E and 5F). This indicates that the expression of p53 and Azurin increased the extent of apoptosis in treated non-responder rats. In Figure S2, DAB staining of tumor from a treated non-responder rat showed increase in p53 levels (expressed by carrier) and an influx of activated microglia (innate immune response) near the carrier injection site, which coincided with increased PARP (greater apoptosis) and decreased ki67 (less cell proliferation) staining. However, increased ki67 was observed in the region surrounding the carrier injection site.

**Figure 6:**
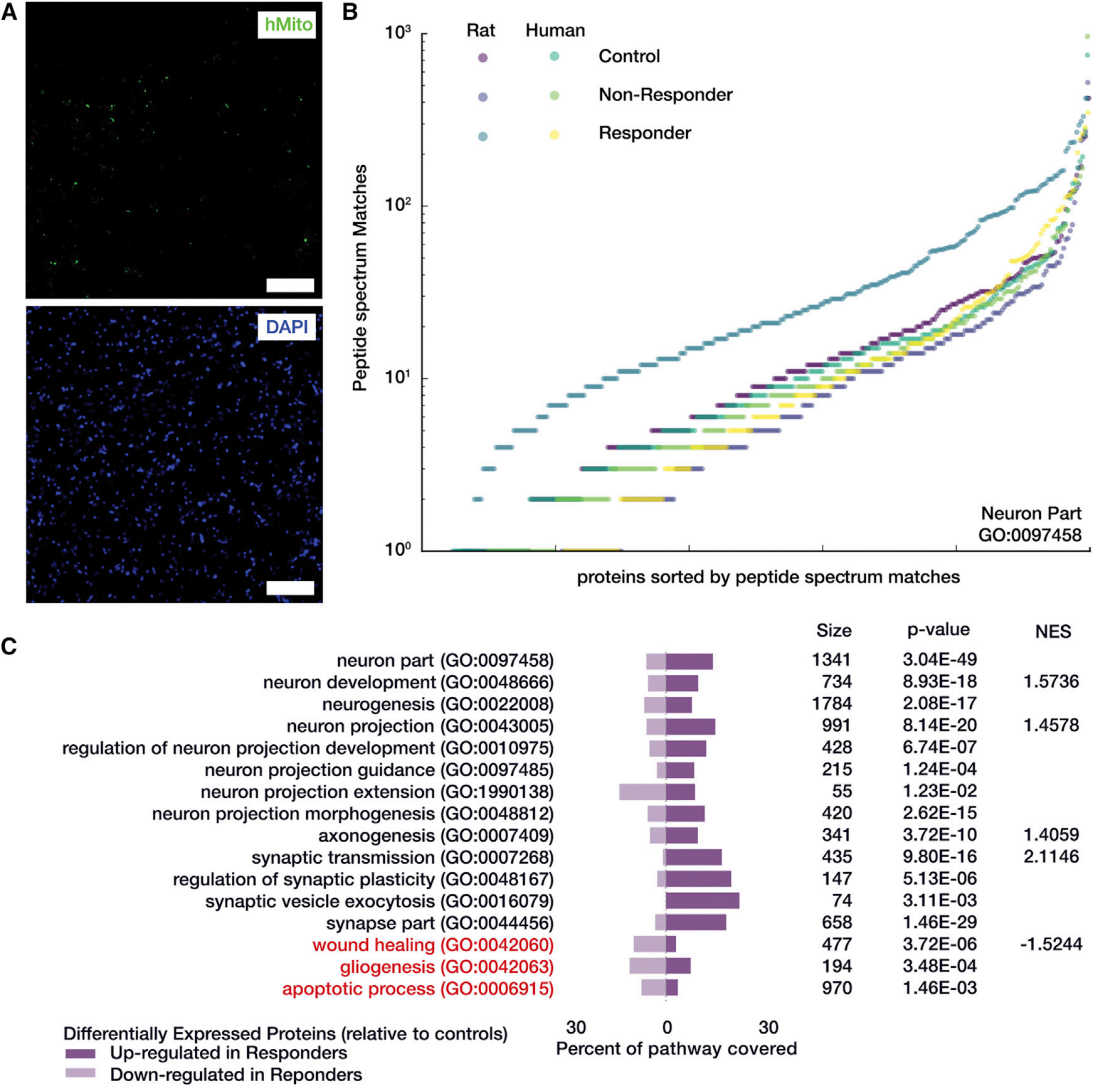
Proteomic Assessment of Bacterial Carrier Responding Cohort (A) Human mitochondria antibody (green, top) and DAPI (blue, bottom) for treated responders showed a complete loss of GBM cells (cf. Figure 5). Images were contrast-enhanced digitally to improve visibility of low fluorescent signals. Scale bar, 100 μm. (B) Individual peptide spectrum match counts for proteins in the Neuron Part (GO: 0097458) ontology that were also measured by proteomics. Protein measurements are split by both outcome condition and species of protein assignment and sorted for cumulative display. Proteins that were measured as rat in the responder cohort were substantially more up-regulated overall than all other conditions. (C) Curated list of pathways over-representation analysis that emphasize a pro-neural protein signature. Bar graphs indicate the number of differentially expressed proteins that were measured via proteomics and also overlap with the related ontology and whether they were up- or down-fold relative to the control cohort. NES is the normalized enrichment score from gene set enrichment analysis used in this study, where a positive score indicates significant enrichment of the pathway in the responder cohort, whereas a negative score indicates a significant down-regulation of the pathway.

One of the treated rats that after carrier inoculation did not show tumor growth per MRI was euthanized early on day 52. The brain sections of this rat showed slightly greater number of tumor cells compared to the ones euthanized on day 104 (Figures S3A–S3C), but the number was severalfold lower compared to control and non-responder rat brain (Figures S3D and S3E). The treated responder brain sections at day 52 showed positive staining for p53, PARP, and TUNEL within the tumor region, indicating apoptotic responses exerted by the carrier (Figures S3A–S3C). This means that, in the treated responders, the tumor population continues to diminish in response to the treatment, and later reaches undetectable levels as time elapses (Figure 6A). PARP and TUNEL expression were not observed in p53-negative tissues including adjacent extratumoral brain tissues.

### Proteomic and Pathway Analyses Show Similar Protein Expression between Both Controls and Non-responders, but Dramatically Different Expression Profile for Responders

A cohort of 12 animals with 6 controls, 3 treated non-responders, and 3 treated responders was used for proteomic analysis to further understand the mechanism of action of these tumoricidal carriers (Figures S4A and S4B). An average of 60,006 peptide spectrum matches (PSMs) per sample were assigned to 4,980 uniquely identified proteins (associated with a unique UNIPROT accession ID) and used for further analyses. Hierarchical clustering using Euclidean distance of the set of PSMs (Figure S4D), showed that the responder group relative to both control and non-responder groups were distinctly clustered, whereas the non-responder and control group were more closely related as two of the controls clustered better with the non-responders than with the other controls.

Statistical analysis was performed and used to define differentially expressed (DE) proteins by filtering for ±2-fold change between conditions and applying a false-discovery rate (FDR) threshold at 0.05. In total, 2,459 differentially expressed proteins were found between the responder and control groups, 2,027 from the responders and non-responders, but only 186 proteins were differentially regulated between the non-responder and control groups (Figure S4C). Table S3 shows the top 20 up- and down- regulated of these proteins between each condition pair. (For complete dataset, please see Data S1, S2, and S3.) The Euler diagram in Figure S4E depicts how these DE proteins group among conditions and whether they were relatively up- or down-regulated for each condition pair clearly showing a large overlap in proteins that are differentially expressed in responders relative to both control and non-responders. The smallest subsets were for non-responders relative to controls, with only eight up-folded DE proteins in total.

To determine which protein signaling pathways were differentially expressed between treatment conditions, pathway overrepresentation was measured using each of the differentially expressed protein sets. Due to the fact that this study uses a xenograft model and the protein accessions were assigned parsimoniously without guarantee of correct species being attributed, this analysis opted to use the related gene symbols instead of protein accessions and performed the lookups against human databases only. Human databases are more thoroughly annotated than those for rat model used in this study, and therefore more accurate for assessing response of human GBM model. The search elicited 284 significantly (by g: SCS threshold) overrepresented pathways for the non-responder:control condition pair, 2,172 for responder:control, and 2,347 for responder:non-responder.

Further inquiry into differential pathway expression was determined using gene set enrichment analysis against the Gene Ontology and Reactome datasets. This was performed using the log2 fold change for all measured proteins between each condition pair as the pre-ranked metric. The five highest and lowest normalized enrichment scores for each condition pair are shown in Table S4. Between the non-responder:control condition pair, only 13 gene sets were found to be significantly enriched in non-responders with an FDR of < 25%, and 8 were significantly enriched in controls. For the responder:control pair, 185 gene sets were significantly enriched in responders and 255 in control. Finally, for responder:non-responder, 146 enriched in responders and 132 in non-responders. These significantly enrichment networks for responder:control and non-responder:control shown in Figure S5. Due to the large distinction between responders and controls, the enrichment map for responders (Figure S5A) is vast. Apparent are groupings of positive enrichment associated with mitochondrial function and biogenesis, transmembrane transport, and neuronal signaling, as well as nervous system development. The connectivity of negatively enriched pathways for responders is less clear, likely due to the overall decrease in tumor cells present in these samples. If there were a significant number of tumor cells present, a diverse positive enrichment in pathways for metabolic processes would be evident. Instead, in the responder network, a negative enrichment for a number of pathways related to protein translation and cell cycle processes was seen. Of the few significantly enriched pathways in non-responders (Figure S5B), most were associated with either ribosomal function, or protein folding and assembly.

### Treated Responders Lack Indication of Residual GBM Tissue and Are Enriched with Pro-neural Proteins

In the treated rats that were euthanized after more than 100 days, only a sparse population of dispersed tumor cells was observed in brain sections stained with an anti-mitochondria antibody specific for human cells (Figure 6A). This is a dramatic contrast to the other outcome cohorts, as the tumor population from both the control (Figure S3D) and treated non-responder (Figure S3E) rats was dense with positive human mitochondria staining.

Many neuro-related pathways were found in both the overrepresentation and enrichment analyses for responders. In particular, the ontology for Neuron Parts (GO: 0097458) showed considerable increase in expression in the responders for peptides that were parsimoniously assigned to rat proteins (Figure 6B). Figure 6C shows several other neuronal pathways, all significantly overrepresented by the DE proteins in responders relative to controls. All but the neuron projection extension pathway showed a clear bias for up-regulated proteins. The gliogenesis pathway was also significantly overrepresented; however, the split between DE up- and down-regulation was mixed, likely indicating both a loss of GBM-related proteins and renewal of a healthy glial cellular milieu. Wound healing (GO: 0042060) and apoptotic processes (GO: 006915) were both biased for down-regulation in the responders, further indicating a renewed neural environment.

### Protein Analysis Showed Differential Regulation of Both Pro- and Anti-apoptotic Proteins between Control and Non-responder Rats

**Figure 7:**
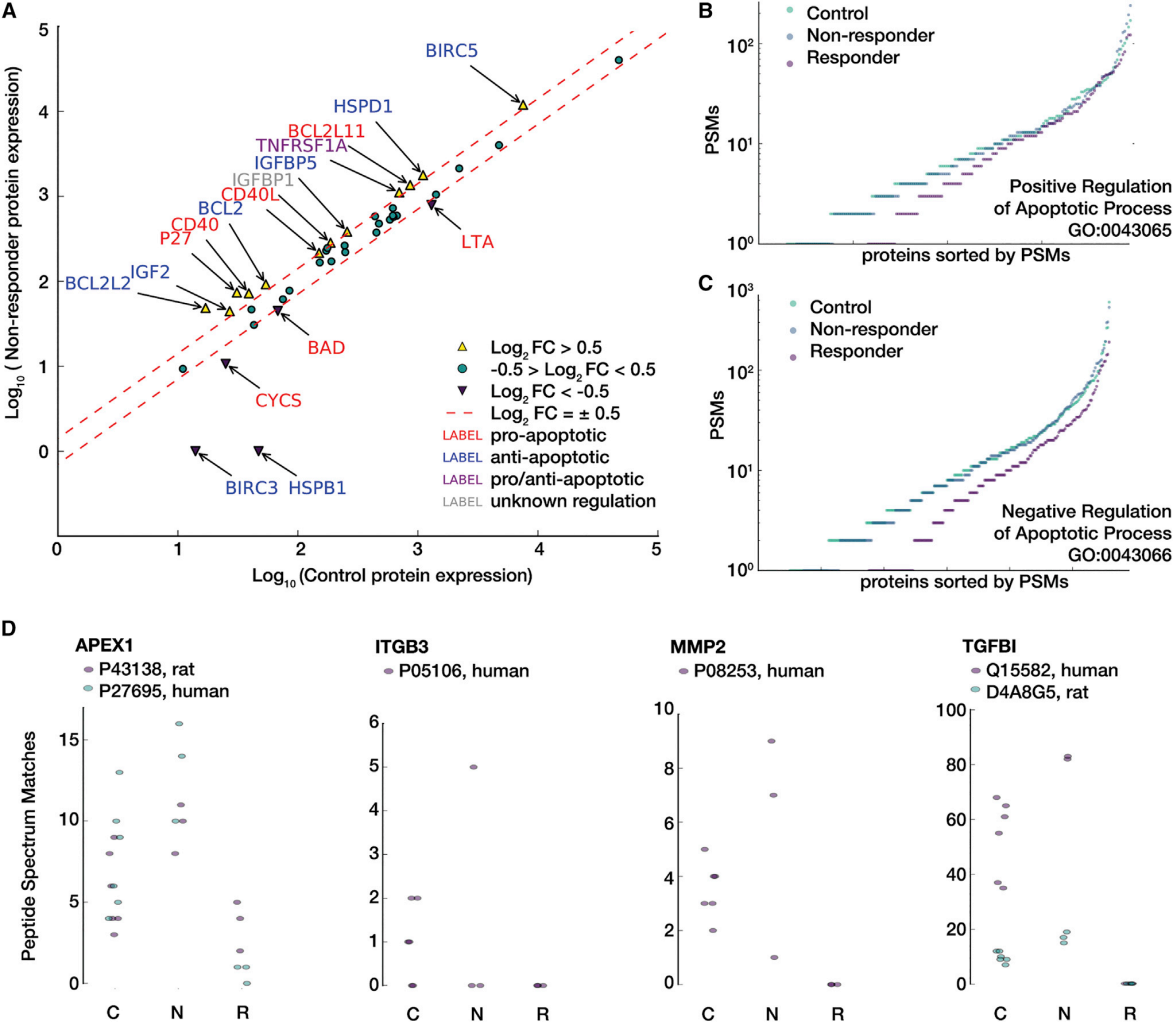
Proteomic Assessment of Bacterial Carrier Non-response (A) Results from G-series apoptosis array to check for levels of apoptotic proteins in the tumor lysate from control and treated non-responder rats. Proteins with a ±2-fold change are annotated and colored depending on their regulation of apoptosis. Again, there appears to be no dramatic difference between the conditions, and for the majority of those proteins that are distinct, no clear positive or negative apoptotic regulation is clear. Shown next are individual peptide spectrum match counts for proteins in the ontologies for (B) positive (GO: 0043065) and (C) negative (GO: 0043066) regulation of apoptosis that were also measured by proteomics. Protein measurements are split by both outcome condition and sorted for cumulative display. Proteins that were measured in the responder cohort showed substantially lower expression than in controls and non-responder, both of which were relatively indistinguishable when looking at these overall pathway responses. (D) Peptide spectrum matches for posited therapy resistance protein markers. Up-regulation of APEX1, MMP2, and TGFBI, and down-regulation of ITGB3 are consistent with a drug-resistant phenotype in glioma. C, control; N, non-responders; R, responders.

In order to check for differences in the type and relative levels of apoptotic proteins in the tumors of non-responders and controls, a G-series apoptotic antibody array analysis was performed on the tumor lysate (RayBiotech AAH-APO-G1). Statistical analysis was not performed, as the biological replicates were pre-pooled before analysis; instead, relative fold change in expression is reported (Figure 7A). Twelve of these proteins were more than 2-fold up-regulated, whereas 5 were more than 2-fold down-regulated in non-responders relative to controls.

A BIOGRID^55^ protein-protein interaction neighborhood was generated for the antibody array proteins and rearranged to emphasize connectivity with respect to p53. This network was colored from relative protein expression and showed that p53-associated signaling in non-responders relative to controls (Figure S6A, proteomic and array data averaged) is dramatically different and not consistently up- or down-regulated. However, a comparison of responders relative to controls (Figure S6B, proteomic data only) exhibited a general down-regulation of all p53-associated proteins.

Figure S7 shows coverage of measurements used in this study on the Kyoto Encyclopedia of Genes and Genomes (KEGG)^56^ canonical p53 Signaling (HAS: 04115) (Figure S7A) and apoptosis (HSA: 04210) (Figure S7B) pathways, colored using average non-responder fold change relative to controls, using proteomics and western array data. Moreover, when comparing the full set of proteins that either positively or negatively regulate apoptosis (Figures 7B and 7C), non-responders and controls do not appear to be distinct; the related expression in responders, however, is generally lower when looking at these broad regulatory ontology sets.

## Discussion

Facultative anaerobic bacteria such as *S. typhimurium* have been previously shown to preferentially multiply in hypoxic and necrotic regions within a tumor.^24^ This active tumor-targeting potential of bacteria has been previously exploited to express tumoricidal proteins that can induce apoptosis and suppress tumor growth in different cancer types.^57^ The particular novelty of this study is the use of *S. typhimurium* to deliver a tumoricidal combination therapy of p53 and Azurin that synergistically induce apoptosis in an intracranial rat model of aggressive GBM tumor in the absence of an adjuvant therapy. In GBM, drug diffusion is a major impediment to successful treatment, and the motile phenotype of the bacteria is advantageous in that active delivery through the tumor can be achieved without any external pressure or chemical gradient, thus overcoming the difficulties encountered with CED. Additionally, use of bacteria as a carrier for plasmid-mediated expression of proteins affords improved programmability and sustained drug delivery compared to conventional gene therapies.

*S. typhimurium* is a known intracellular pathogen, and in particular, the VNP strain has been shown to express proteins in host tissues^53^, ^58^, ^59^ (also observed in Figure 3). Once a VNP carrier invades the host cell, it can express Azurin, which gets secreted outside the bacterial cell into the host cytosol (due to its N-terminal secretion signal sequence) where it can exert its apoptotic activity or protect endogenously synthesized or bacterially expressed p53. In case of p53, although it lacks a secretion signal, p53 secretion was observed in vitro (Figure 1C) by an unknown mechanism. Furthermore, we observed that the levels of p53 in the host tissue that has the carrier is greater than in control rats (Figures 5A and 5B), indicating successful p53 expression and release. Once released, we hypothesize that p53 can express its apoptotic action within the cytosol by up-regulating proteins such as Bax that creates pores in mitochondrial outer membrane and starting the caspase cascade. Because we have used carriers to express wild-type p53, and this protein has an intact proline-rich domain and nuclear localization signaling domain, we hypothesize that cytosolic p53 can make its way into the nucleus in order to initiate additional apoptotic pathways.

Coincident expression of p53 and Azurin was required for a survival benefit in U87mg-xenografted rats (Figures 2B, S1A, and S1B). The inability of p53 alone to induce apoptosis in U87mg cells is consistent with previous work^60^, ^61^, ^62^ showing that, although U87mg cells natively express wild-type p53, they are apoptotic resistant to induction of exogenous wild-type p53 alone. Furthermore, p53 is a labile protein and by itself is subject to ubiquitination and proteasomal degradation, with a half-life of approximately 25–30 min if left unprotected.^46^, ^47^, ^48^ Hence, Azurin was co-expressed with p53 in order provide the requisite stabilization.

To mitigate concerns over the use of tumoricidal carriers and their potential impact on healthy tissues and host response, we have opted for an avirulent carrier, *S. typhimurium*, VNP 20009, and in addition, engineered expression of apoptotic proteins to be regionally limited. Azurin- and p53-encoding genes were cloned downstream of a hypoxic promoter, *pflE*, which has been shown in previous studies to offer dual advantages: (1) it is a microaerobically induced, which means that is greatly induced under hypoxic conditions compared to normoxic conditions; and (2) this promoter has been shown to turn “on” genes only within tumors and not in other tissues,^52^, ^53^ thus avoiding unintended toxicity to healthy tissues.

The prolonged preferential presence of the bacteria within the tumor, and the hypoxia-dependent production of p53 and Azurin allow sustained expression of the proteins of interest in the region of interest. As observed in treated non-responders (Figure 3), the carrier localized mainly to the tumor with minimal presence in healthy tissues, indicating that the nutrient-rich, immunosuppressed microenvironment of the tumor provides a preferential location for the carrier to reside and multiply.^24^ Even with a baseline p53 expression under normoxic conditions (Figure 1C), p53 expression and p53-mediated apoptosis were not observed in the adjacent healthy brain tissues (Figures S3A–S3C). Importantly, even with this sustained presence of a foreign organism, treatment with the carrier does not appear to directly compromise animal safety, as none of our surviving animals showed bacteria-associated symptoms of diminished health or systemic toxicity as indicated by TNF-α levels in the blood (Figure 4).

Previous studies have shown that these carriers are quickly cleared from the bloodstream either due to the phagocytic action of polymorphonuclear leucocytes, or via the reticuloendothelial system of the liver and the spleen, thus alleviating the overall infectious state of the animal.^63^, ^64^, ^65^ In addition to the multiple host mechanisms enabling clearance of the bacteria, the carriers can also be cleared by administration of an antibiotic, such as tetracycline and enrofloxacin.^23^

Our compiled in vivo tumor implant studies demonstrate that, in 19% of treated rats, the tumor growth was retarded and the rats survived for more than 100 days (Figure 2B). In the treated rats, the response was discretely either therapeutic or not. Decrease in tumor volume was only seen in the survival cohort, and the indication of tumor regression was tied directly to the measurement following either the first or second injection of carrier. The fact that some animals did not show a therapeutic response until after the second injection is important to note, as it suggests that increasing the number or altering the schedule of injections may be sufficient to produce an improved response rate.

In the responding animals, we found only trace evidence of GBM cells histologically (Figure 6A). Proteomic analysis clearly showed a restored neural environment as evident by the numerous pro-neural pathways that were either overrepresented or enriched in responders relative to both treated non-responders and controls (Figures 6B and 6C). This is a strong indication that there is a significantly large representation of neuronal cells in the responder tissue, likely due to a lack of GBM cells. The global protein signatures for responders are also very distinct from both the non-responders and controls (as seen in terms of PSM distance; Figure S4D). In contrast, when using this same distance metric and even when looking at the signaling pathways for positive and negative regulation of apoptosis (Figures 7B and 7C), non-responders and controls are essentially indistinct. This is confirmed by more sensitive antibody-based measurement of particular apoptosis-associated proteins seen in Figure 7A. Differentiating these proteins by pro- and anti-apoptotic function also elucidates no clear trend in overall apoptotic regulation. For instance, BIRC3 and HSPB1, both anti-apoptotic proteins are substantially down-regulated in non-responders, but so is CYCS, a key p53-mediated, pro-apoptotic initiator. However, this ambiguous response is not necessarily surprising, given that we observed increased TUNEL-positive expression (Figures 5E and 5F) in the non-responders (indicating pro-apoptotic processes) yet did not see tumor regression overall in those animals via both MRI-measured tumor volume (Figures 2C and 2D) and in terms of survival (Figure 2B).

When looking specifically at the p53-mediated protein network, however, there are considerable differences between the non-responders and controls. To explore the regulation of apoptosis as centered around p53, a BIOGRID^55^ protein-protein interaction neighborhood was generated for these apoptosis-associated proteins that were colored using the combined array and proteomic data for non-responders relative to controls (Figure S6A). Within this network, there was a dramatically different p53-associated signaling in non-responders relative to controls, suggesting that there is a differential regulation of p53 in these animals. At least, this seems to indicate a perturbation in p53-associated signaling in response to the carrier even if it is not robust for pro-apoptosis. In contrast, the responders appear to be generally down-regulated across the p53 neighborhood relative to controls (shown using only proteomics data in Figure S6B) likely due to the lack of active carriers once the tumor is depleted.

Last, we investigated the protein data for indications of compensatory mechanisms, other than those that are directly anti-apoptotic, that exhibited a drug resistance-like response in non-responders. Although many key glioma drug resistance markers were not detected (e.g., MDR1, MRP, LCN2), Figure 7D shows the peptide spectrum matches for four proteins that were detected via proteomics of particular note. APEX1, an endonuclease associated with gliomagenesis that is known to be correlated with reduced therapeutic efficacy in glioma,^66^ showed an overall increased expression in non-responders. MMP2, a collagenase that can be transcriptionally activated by p53^67^ and is associated with enhanced tumor growth in glioma^68^ showed a dramatic increase in two of three non-responders. Both accession identities for TGFBI, another indicator of glioma invasiveness^69^ that may be secreted in drug-resistant glioma,^70^ showed relative increase in expression. Last, ITGB3, an integrin component that is known to be down-regulated in drug-resistant gliomas,^69^ was undetected in two of three non-responders.

Even with this detailed proteomic analysis, no solely pro- or anti-apoptotic signal in the non-responders could be resolved. The lack of spatial discrimination and temporal resolution of the protein analyses may have contributed to the ambiguous evidence of apoptotic regulation. Temporal resolution was limited mainly due to lack of non-invasive measurement of tumor progression other than MRI and no direct measurement of apoptotic signaling in situ until after the animal is euthanized. Furthermore, without clear predictive outcomes, we could not reliably sacrifice an animal early knowing that the animal would be in the survival cohort or not. We were, however, able to sacrifice one suspected responder early at day 52, but this was done in a late cohort after we had established that animals surviving past day 40 were likely responders. With respect to spatial factors, treated non-responder status could be due to the phenotypic heterogeneity of the cell population as the tumor grows in its immunosuppressed environment and adopts a number of intrinsic and acquired mutations, with which tumors easily develop resistance to a number of therapeutic agents.^71^ This may explain why, although in some parts of the treated non-responder rats the tumor underwent apoptosis, in other parts the tumor grew rampantly, resulting in increased tumor growth. The increased rate of tumor cell proliferation may have also impacted the extent of apoptosis as evident in Figure S2. Although ki67 staining is decreased directly near the carrier injection site, in the surrounding region, the tumor cells continued to grow. This indicates that, even though a therapeutic response is seen, it may be constrained regionally and can be overcome by the rate of tumor proliferation.

In conclusion, 19% of treated rats showed clearance of aggressive GBM. The treated non-responders demonstrated tumoricidal action via the engineered bacterial carriers as evident from multiple lines of analyses including immunohistochemistry and proteomics. The proteomic signatures suggest a complex response to the carrier therapy in non-responder animals and incontrovertible evidence for tumor clearance in the treated responders. Importantly, the carriers were safe, with no evidence of any systemic toxicity. The evidence of apoptotic action of p53 and Azurin suggest that motile carriers represent versatile approach to expressing multiple tumoridical proteins in brain tumors.

## Materials and Methods

### Construction of Azurin- and p53a-Expressing Carrier under the Control of Hypoxic Promoter

The *pflE* promoter that is upstream of the gene encoding pyruvate formate lyase in *S. typhimurium* was amplified from genomic DNA of this bacterium using primers PflE-F and PflE-R, and cloned in place of the lacZ promoter in pDSRed2 plasmid (Clontech). This was followed by deleting DSRed2 gene and replacing it with either gene encoding p53 (using primers p53-F and p53-R) or Azurin (using primers Azurin-F and Azurin-R). Source of p53 was pUNO1hp53a plasmid (InvivoGen), and that of Azurin was genomic DNA of *Pseudomonas aeruginosa* PAO1. The pPflE-p53 and pPflE-Azurin cloned plasmids were individually transformed into chemically competent *S. typhimurium* strain VNP 20009 (ATCC 202165). Positive transformants were confirmed by PCR. Primer sequences are given in Table S1. Expression of p53 and Azurin from aerobically and anaerobically grown bacteria (carrier) harboring pPflE-p53 or pPflE-Azurin plasmid was confirmed by western blotting or Mass Spec, respectively. Expression of p53 was also confirmed by ELISA.

### Tumor Implant Protocol in Athymic Nude Rats followed by Intracranial Injection of Carrier Expressing p53 and Azurin

All animal procedures were conducted in accordance with Georgia Tech’s Institutional Animal Care and Use Committee (IACUC). Adult male athymic RNU rats from Charles River laboratories were anaesthetized with inhaled isoflurane (2%–2.5%) and fit onto a stereotaxic device. A burr hole was drilled 2.0 mm lateral and 2.0 mm anterior to bregma to a depth of 2.0 mm in the left cortex. A 26G Hamilton needle was used to inject 80,000 human U87 MG GBM cells (ATCC HTB-14) into the drilled hole in a total volume of 5 μL at a rate of 1 μL/min. The drilled hole was closed using bone wax and the skin flap of the brain sutured. Upon detection of a small tumor in MRI after a week, one set of rats was injected at the site of tumor implant (ic) with 5 μL of carrier strains expressing p53 and Azurin grown individually and mixed 1:1 just before the injection (approximate count of each strain, 5 × 10^6^ cells). This set of treated rats were injected (ic) one more time a week later with the same two strains. The control rats were injected with 5 μL of saline (ic). MRI was done weekly until the time of euthanization.

### MRI Imaging to Check for Tumor Formation

The athymic nude rats were anaesthetized and placed in a Bruker Pharmascan 7-T (Bruker BioSpin MRI) operating with the ParaVision software with a 38-mm quadrature-detection volume coil as head coil. The animal was anaesthetized using 2% isoflurane and placed in a home-built cradle, allowing the easy placement of the animal’s head within the MRI coil. The rapid acquisition of high quality T2 weighted images was achieved using the rapid imaging with refocused echoes (RARE) sequence (RARE factor, 6; effective echo time, 36 ms; repetition time [TR], 4,200 s; two averages per scan; total acquisition time, 6 min). A slab of 40 transversal slices was recorded using a field of view of 40 mm × 40 mm with a 256 × 256 matrix and a slice thickness of 0.5 mm. This slab was aligned to cover the injection site of the tumor cells using a pilot scan, which was recorded immediately before the acquisition of the RARE images. MRI was done every week following tumor implant to check for tumor growth or regression.

### Tissue and Blood Collection for Protein Work, ELISA, and IHC

All euthanization and tissue-harvesting procedures were conducted in accordance with Georgia Tech’s Institutional Animal Care and Use Committee (IACUC). Animals that showed clinical signs of illness were anaesthetized using ketamine (50 mg/kg), xylazine (10 mg/kg), acepromazine (1.67 mg/kg) cocktail. Then they were transcardially perfused with either sterile PBS (for protein work and ELISA) or with 4% paraformaldehyde (for IHC). The brain was harvested and transferred into liquid nitrogen or 10% buffered formalin, depending on tests to be performed. Some part of the tumor tissue (from control and treated non-responders) and part of the brain tissue (from treated responders) were sent for proteomics to Emory Core Proteomics Lab. The liver and spleen were harvested and stored in 10% buffered formalin. Blood from the heart was collected as a terminal procedure by using cardiac puncture technique. Serum was separated from the blood and used for ELISA.

### Fluorescent in Situ Hybridization to Check for Presence of Carrier

To check for presence of carrier in brain, liver, and spleen, the tissue was sectioned to 7-μm thickness. The sections were permeabilized and hybridized with fluorescently labeled probe (Table S1) that binds specifically to the 23S rDNA of *S. typhimurium* at 46°C for 2 hr using a protocol as previously described.^72^ Sections were then counterstained with DAPI. Images were viewed using the Zeiss LSM 700-405 confocal microscope.

### Recovery of VNP 20009 from Immunocompetent Sprague-Dawley Rat Brains

Rats (n = 3) were injected intracranially (left cortex) and intravenously (tail) with VNP 20009. These rats did not have tumor inoculation. After 15 days, rats were euthanized and brains were homogenized and streaked on bacterial plates. Following 24-hr incubation, the colonies were counted.

### ELISA to Check for Levels of TNF-α in Serum as an Indicator of Systemic Toxicity

Blood collected from heart was centrifuged at 1,000 × *g* for 10 min to separate the serum. TNF-α level in the serum was measured using anti-TNF-α ELISA kit as per the manufacturer’s instructions (BD Biosciences).

### Immunohistochemistry to Check for p53, Cleaved PARP, TNF-α, ki67, and CD68

To check for levels of p53 expression and cleaved PARP (indicator of apoptosis), the tissues were sectioned to 14-μm thickness, fixed with 4% paraformaldehyde, and blocked with blocking buffer for 1 hr. This was followed by treating with anti-p53 (Sigma; P 8999) or anti-cleaved PARP (Thermo Fisher Scientific; 44-698G) antibodies overnight in blocking buffer. On subsequent day, the sections were treated with fluorescent secondary antibodies. Sections were imaged on a Zeiss LSM 700-405 confocal microscope.

To check for levels of TNF-α (pro-inflammatory cytokine), ki67 (cell proliferation), CD68 (activated microglia), and cleaved PARP (apoptosis), the tissues were sectioned in 4- to 5-mm blocks and submitted as individual cassettes for paraffin embedding at Emory Winship Pathology Core Lab. Tissues from paraffin-embedded blocks were sectioned at 5-μm thickness. IHC was performed using DAB chromogenic kit (Wako) following the manufacturer’s protocol. Antibodies used were anti-TNF-α (Boster Biologics; PA 1079), anti-p53 (Sigma; P8999), anti-cleaved PARP (Thermo Fisher Scientific; 44-698G), anti-ki67 (Thermo Fisher Scientific; MA5-15690), and anti-CD68 (AbD Serotec; MCA 341R). Whole-slide scanning was done using Hamamatsu’s Nanozoomer 2.0 HT.

### TUNEL for Brain, Liver, and Spleen Sections to Check for the Extent of Apoptosis

In Situ Cell Death Detection kit was used to label nicked 3′ ends with TMR-conjugated dUTP in order to detect apoptosis in tissue sections as per the manufacturer’s instructions (Sigma).

### Tissue Homogenization prior to Liquid Chromatography-Tandem Mass Spectrometry at Emory Proteomics Core Lab

Brain tissue was individually homogenized in 300 μL of urea lysis buffer (8 M urea, 100 mM NaHPO_4_, pH 8.5) which had 3 μL (100× stock) of HALT protease and phosphatase inhibitor cocktail (Pierce). Homogenization was performed using a Bullet Blender (Next Advance) according to manufacturer protocols. Briefly, each tissue piece was added to urea lysis buffer in a 1.5-mL Rino tube (Next Advance) harboring 750-mg stainless-steel beads (0.9–2 mm in diameter) and blended twice for 5-min intervals at 4°C. Protein supernatants were transferred to 1.5-mL Eppendorf tubes and sonicated (Sonic Dismembrator; Fisher Scientific) three times for 5 s with 15-s intervals of rest at 30% amplitude to disrupt nucleic acids and subsequently vortexed. Protein concentration was determined by the BCA method, and samples were frozen in aliquots at −80°C. Protein homogenates (100 μg) were diluted with 50 mM NH_4_HCO_3_ to a final concentration of less than 2 M urea and then treated with 1 mM dithiothreitol (DTT) at 25°C for 30 min, followed by 5 mM iodoacetamide (IAA) at 25°C for 30 min in the dark. Protein was digested with 1:100 (w/w) lysyl endopeptidase (Wako) at 25°C for 2 hr and further digested overnight with 1:50 (w/w) trypsin (Promega) at 25°C. Resulting peptides were desalted with a Sep-Pak C18 column (Waters) and dried under vacuum.

### Statistical Analysis

IHC comparisons were done in Prism 6 (for OSX; GraphPad Software) on pre-averaged biological replicates using a t test with Welch’s correction for two group comparisons or ANOVA with Tukey multiple comparisons for comparisons across three groups (alpha = 0.05). Survival analysis was also done in Prism, using log rank (Mantel-Cox) to compare median survival across groups.

### Liquid Chromatography-Tandem Mass Spectrometry Analysis at Emory Proteomics Core Lab

Brain-derived peptides were resuspended in peptide 100 μL of loading buffer (0.1% formic acid, 0.03% trifluoroacetic acid, 1% acetonitrile). Peptide mixtures (2 μL) were separated on a self-packed C18 (1.9 μm; Dr. Maisch) fused silica column (25 cm × 75 μm internal diameter [ID]; New Objective) by a Dionex Ultimate 3000 RSLCNano and monitored on a Fusion mass spectrometer (Thermo Fisher Scientific). Elution was performed over a 120-min gradient at a rate of 300 nL/min with buffer B ranging from 3% to 80% (buffer A: 0.1% formic acid in water; buffer B: 0.1% formic in acetonitrile). The mass spectrometer cycle was programmed to collect at the top speed for 3-s cycles. The mass spectrometry (MS) scans (400–1600 *m/z* range, 200,000 AGC, 50-ms maximum ion time) were collected at a resolution of 120,000 at *m/z* 200 in profile mode and the HCD MS/MS spectra (2 *m/z* isolation width, 30% collision energy, 10,000 AGC target, 35-ms maximum ion time) were detected in the ion trap. Dynamic exclusion was set to exclude previous sequenced precursor ions for 20 s within a 10-ppm window. Precursor ions with +1, and +8 or higher charge states were excluded from sequencing.

Spectra were searched using Proteome Discoverer 2.0 against human, rat, and *Salmonella* concatenated Uniprot database (134,326 target sequences). Searching parameters included fully tryptic restriction and a parent ion mass tolerance (±20 ppm). Methionine oxidation (+15.99492 Da), asparagine and glutamine deamidation (+0.98402 Da), and protein N-terminal acetylation (+42.03670) were variable modifications (up to three allowed per peptide); cysteine was assigned a fixed carbamidomethyl modification (+57.021465 Da). Percolator was used to filter the peptide spectrum matches to a false-discovery rate of 1%.

### Proteomic Differential Expression

For proteomics, comparisons between fold changes of the PSMs were used to determine differential expression between the conditions. Data was pre-filtered (using Python; https://www.python.org/; https://www.scipy.org/) to eliminate samples where the PSMs were only non-zero for a single replicate across conditions. The statistical comparison tool QSPEC (v1.3.3)^73^ (nburnin: 2,000; niters: 10,000; normalized: true) was used to compute a z statistic and FDR for each identified protein compared pairwise between conditions. The p values were obtained from the z statistic using R (http://www.R-project.org/). Proteins with a FDR < 0.05 for a particular fold change comparison between conditions were considered statistically significant and treated as DE for that condition-pair.

### Pathway Overrepresentation Analysis

The DE proteins were pre-filtered (using custom Python code) to exclude proteins with a fold change less than 2-fold either up or down. For each condition-pair, gene ontology was performed using g:profiler version: r1536_e83_eg30 (http://biit.cs.ut.ee/gprofiler/).^74^ Gene symbols were used indifferent to species attribution against the *Homo sapiens* dataset. The search included Gene Ontology, KEGG, Reactome, Regulatory Motif, Human Protein Atlas, CORUM, Human Phenotype Ontology, and OMIM databases and used the built-in g: SCS threshold for significance. Default settings were used. QuickGO (https://www.ebi.ac.uk/QuickGO) web service was used for obtaining additional gene ontology information for visualization with Python.

### Pathway Enrichment Analysis

Gene Set Enrichment Analysis (GSEA) Build 0044 (http://www.broadinstitute.org/gsea)^75^ was used to perform pathway enrichment analysis. Pre-ranked analysis was performed against the gene ontology (c5.all.v5.1.symbols.gmt) and Reactome (c2.cp.reactome.v5.1.symbols.gmt) datasets using the log2 fold change for all identified proteins as the ranking scheme for each condition-pair. Default setting were used except the max and min pathway size exclusion criteria were set to 1,000 and 5, respectively. Enrichment map visualization was done using custom Python code and Enrichment Map, v2.1.0,^76^ in Cytoscape, v3.3.0.^77^

### RayBiotech G Series Western Array Blotting to Check Levels of Pro- and Anti-Apoptotic Proteins in Tumor Lysates

Tumor tissue from controls (n = 4) and treated non-responders (n = 4) were pooled in groups of two and homogenized in 1× cell lysis buffer (from Ray Biotech). Cell debris was removed by centrifugation at 1,000 × *g* for 10 min at 4°C. Total protein in the supernatant (lysate) was estimated using the BCA kit (Thermo Scientific). The 1,000 μg/mL lysate obtained from control and treated non-responder rat tumors was tested on G-series Human Apoptosis Array (AAH-APO-G1-4) using manufacturer’s protocol, and analyzed using the RayBio analysis tool. g: Profiler was used to obtain a BIOGRID^55^ neighborhood for the proteins measured on the array. Neighborhood network visualization was reconstructed using Cytoscape, v3.3.0,^77^ and KEGG.^56^

## Author Contributions

N. Mehta and J.G.L. prepared the manuscript and contributed equally to this work. N. Mehta designed and conducted the experiments. J.G.L. contributed proteomic, pathway, and general data and statistical analyses as well as conceptual and experimental design advice. K.P. contributed design and completion of staining and fluorescent in situ hybridization (FISH) assays, as well as broad technical assistance. N. Mokarram contributed conceptual advice, figure illustrations and manuscript revisions. C.K. contributed data quantification and interpretation for tissue histology and ELISA experiments as well as miscellaneous technical assistance. R.V.B. conceived the overall concept, contributed to experimental design, and supervised the project.

## Conflicts of Interest

The authors declare no conflicts of interest.

## Supplemental Information

Document S1. Figures S1–S7 and Tables S1–S4

Data S1. Comma-Separated Value Data for Comparing Fold Change of Treated Non-responders Relative to Controls

Data S2. Comma-Separated Value Data for Comparing Fold Change of Treated Responders Relative to Controls

Data S3. Comma-Separated Value Data for Comparing Fold Change of Treated Responders Relative to Non-responders

Document S2. Article plus Supplemental Information
