# Supplementary material for: Bacterial Carriers for Glioblastoma Therapy
Source: Mol Ther Oncolytics. 2016 Dec 14;4:1–17. doi: 10.1016/j.omto.2016.12.003 (PMC5363759; doi:10.1016/j.omto.2016.12.003)
Supplement: Document S1. Figures S1–S7 and Tables S1–S4 [file mmc1.pdf]

OMTO, Volume *4*

Supplemental Information

Bacterial Carriers for Glioblastoma Therapy

Nalini Mehta, Johnathan G. Lyon, Ketki Patil, Nassir Mokarram, Christine Kim, and Ravi V. Bellamkonda

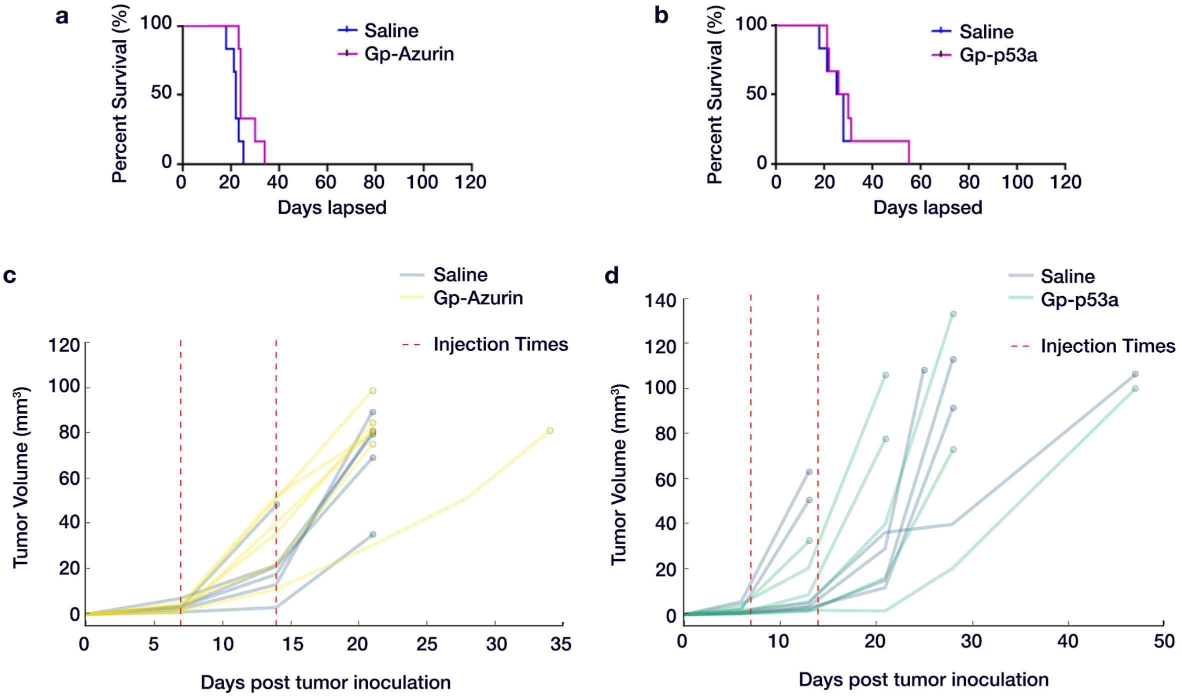

Supplemental Figure S1. Kaplan Meier plot for control (n =6 for each set) and treated rats that were injected with carrier expressing either **(a)** Azurin (n = 6) or **(b)** p53, (n = 6). Results showed no survival in control and treated rat group. *p* value = 0.0263 for Azurin survival plot and *p* value = 0.6721 for p53 survival plot. **(c)** Tumor volumes calculated from MRI images for control (n = 6) and carrier treated rats expressing *only Azurin* (n = 6). Tumor volume increased exponentially in all control and treated rats. **(d)** Tumor volumes calculated from MRI images for control (n = 6) and carrier treated rats expressing *only p53* (n = 6). Tumor volume increased exponentially in all control and treated rats.

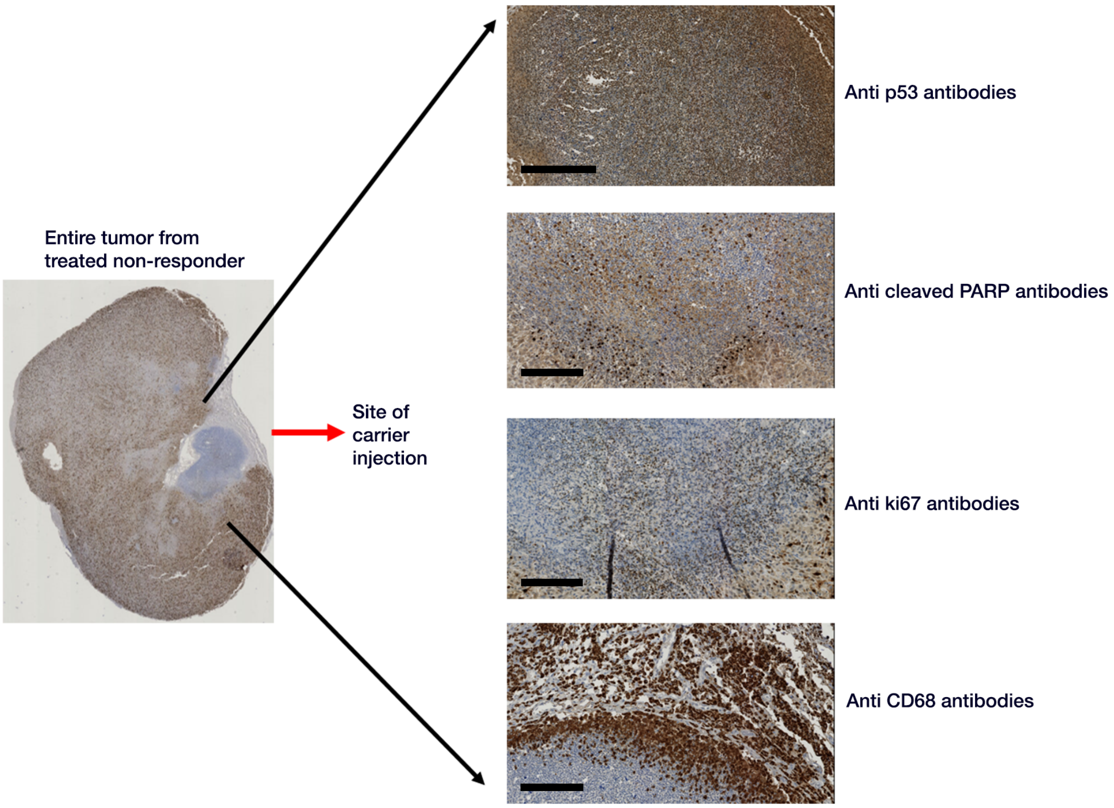

Supplement Figure S2. Multiple responses to apoptotic proteins are observed following carrier injection. Near the site of carrier injection, there is increased expression of p53, increase in cleaved PARP (indicator of apoptosis), decrease in ki67 (indicator for cell proliferation), and an increased influx of activated microglia or CD68 positive cells (indicator of innate immune response). In spite of multiple responses, the tumor of non-responders continued to grow possibly because the rate of tumor proliferation exceeded the rate of apoptosis. Picture shown on left is entire tumor from a non-responder rat stained with anti-ki67 antibodies showing high proliferation rate in most part of the tumor except the site of carrier injection. Scale bars: 200 μm.

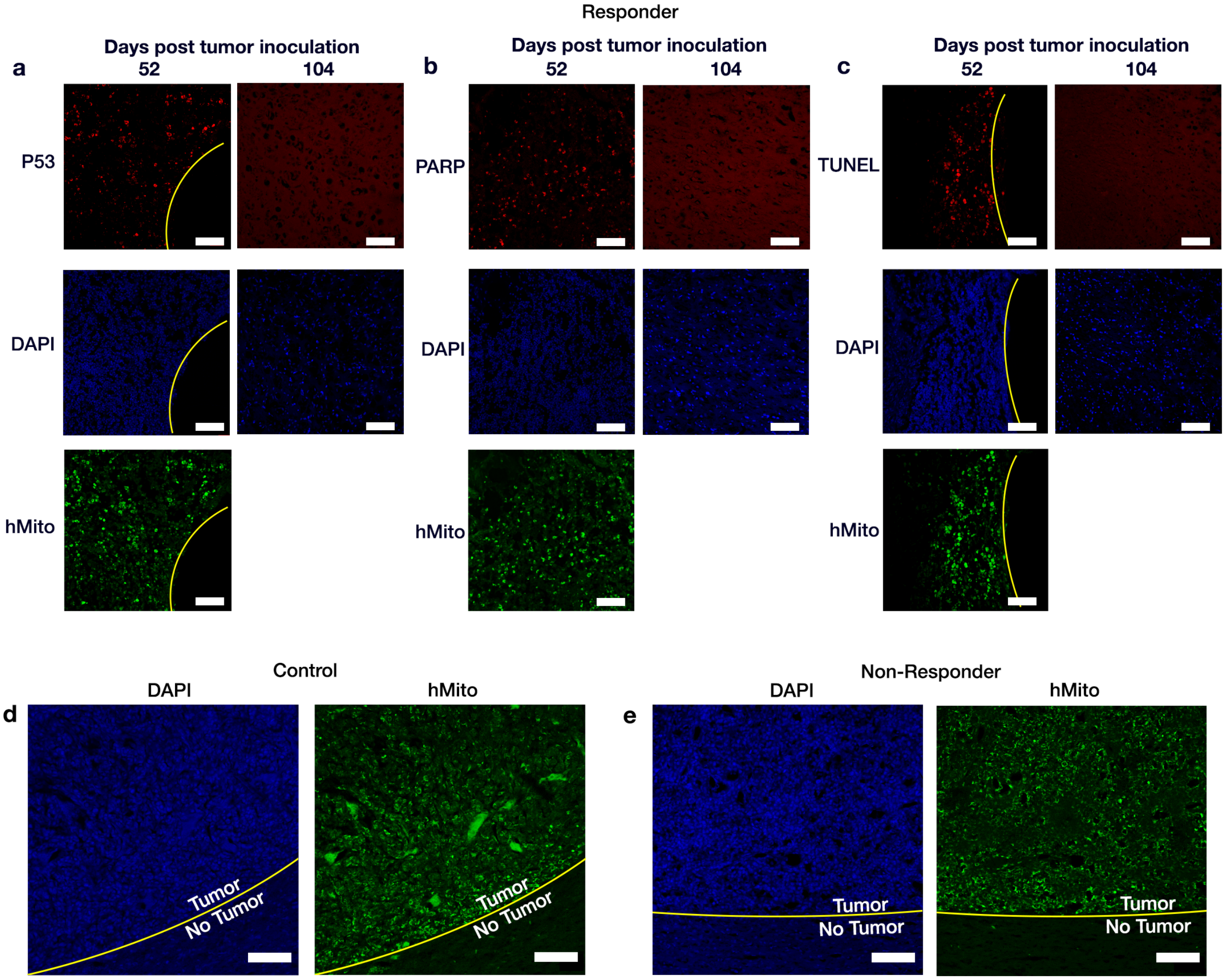

Supplemental Figure S3. IHC images of brain sections from treated responder rats euthanized on day 52 post tumor implant or at day 104 to check for presence of **(a)** p53, **(b)** PARP, or **(c)** TUNEL, each stained red above. Green- human anti-mitochondria antibodies which specifically stains tumor cells, Blue-DAPI. The sections show sparsely dispersed tumor cells (green) on day 52. Right side of yellow line - carrier injection site shows a big gap, presumably the site where tumor cells were originally proliferating. In the 52-day section, lot of p53, PARP and TUNEL staining observed exclusively within tumor region. The adjacent healthy cells did not show TUNEL staining indicating absence of apoptosis in this region. In the day 104 sections, p53, PARP and TUNEL staining was negative. There were no glioma cells in all of the responder tissues euthanized on day 104 (**See Fig 6a**), thus no representative human anti-mitochondrial signal for these conditions. Compared to treated responder rats, the tumor population from **(d)** control and **(e)** treated non-responder rats was dense**.** Yellow lines demarcate intra- and extra-tumoral regions. Scale bars: 100 μm.

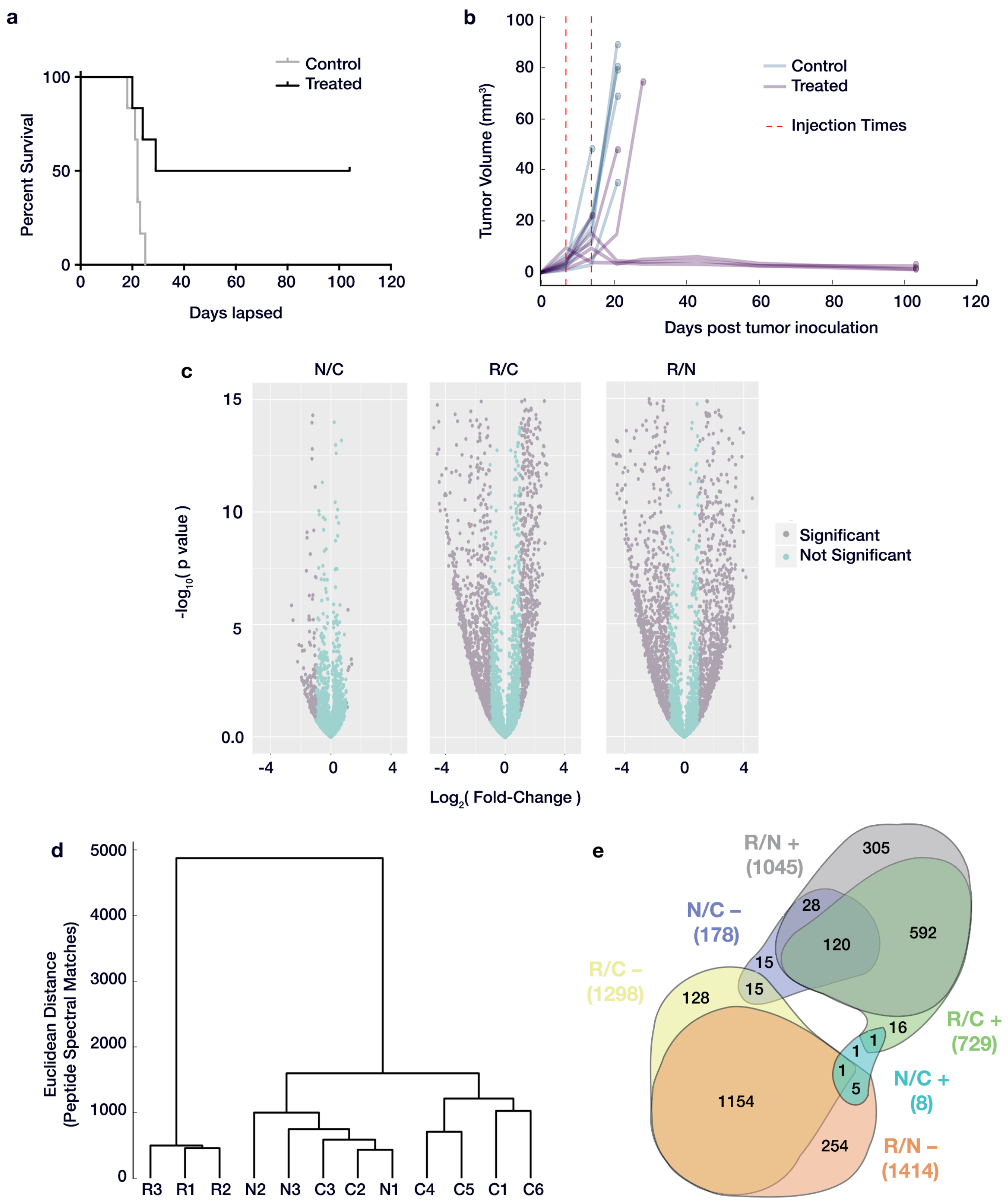

Supplemental Figure S4. (a) Survival analysis and **(b)** tumor volumes for animal cohort used in proteomic analysis (12 animals, 6 control, 3 non-responder, 3 responder). **(C)** Volcano plots depicting significantly differentially expressed proteins. Each dot represents a unique protein (by uniprot accession). Significant threshold was set to a false discovery rate of < 0.05 and a fold change of greater than ±2-fold. (d) Dendrogram

clustering of proteomics samples by Euclidian distance between peptide spectrum matches across all measured proteins. Note the strong clustering of responders (R1-3), while the non-responders (N1-3) and controls (C1-6) are more closely related in their proteomic signature. **(e)** Euler Diagram depicting overlapping membership of differentially expressed proteins across the fold-change pairs and further subset by whether this differential expression was relatively an up- or down- fold change. Numbers in parentheses indicate total number of proteins in that particular differential expression subset. R/C is differentially expressed proteins in responders relative to controls, N/C is non-responders relative to controls, and R/N is responders relative to controls, where the sign indicates either +: up- or –: down- fold differential expression.

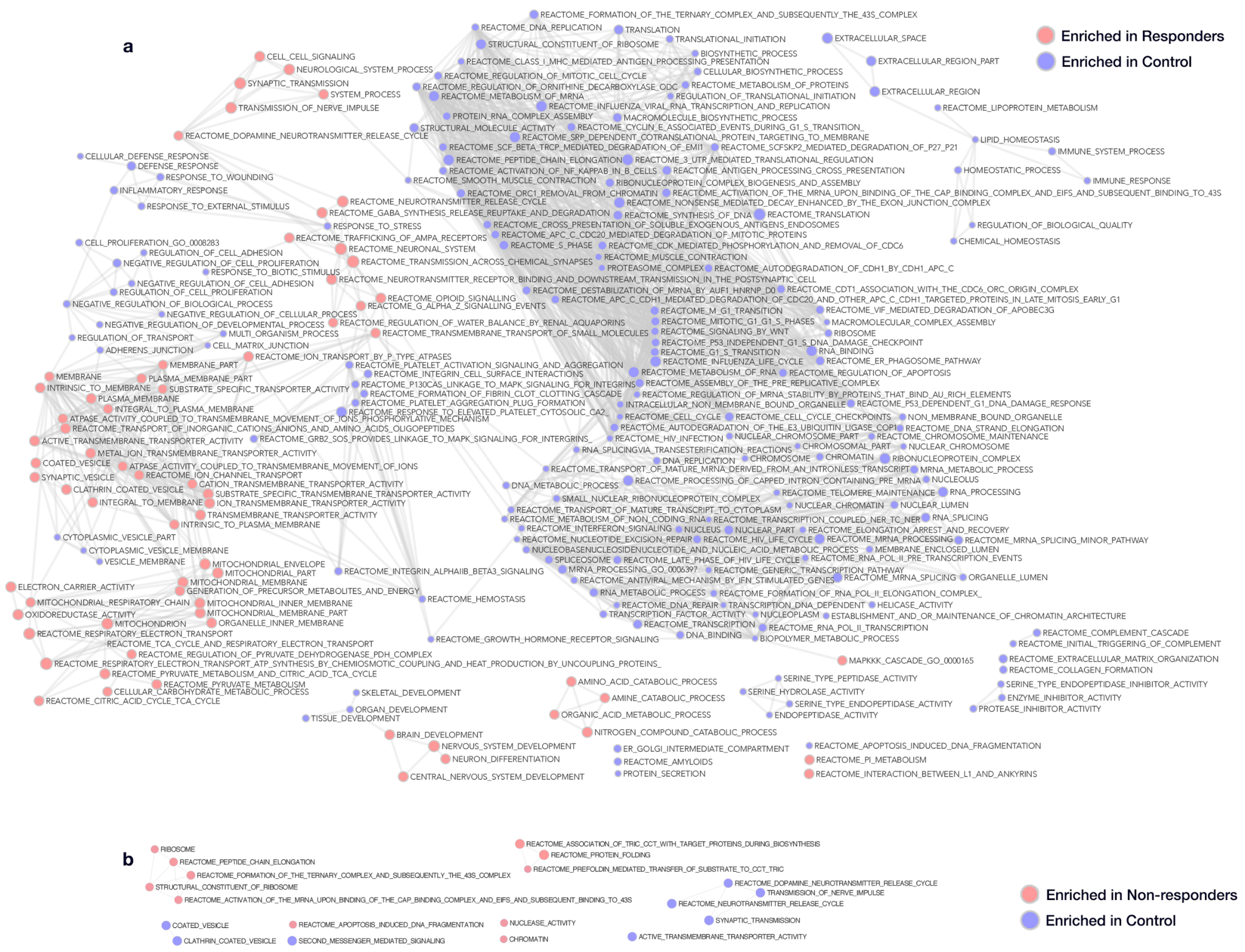
a
Enriched in Responders
Enriched in Control
b
RIBOSOME
REACTOME_PEPTIDE_CHAIN_ELONGATION
REACTOME_FORMATION_OF_THE_TERNARY_COMPLEX_AND_SUBSEQUENTLY_THE_43S_COMPLEX
STRUCTURAL_CONSTITUENT_OF_RIBOSOME
REACTOME_ACTIVATION_OF_THE_MRNA_UPON_BINDING_OF_THE_CAP_BINDING_COMPLEX_AND_EIFS_AND_SUBSEQUENT_BINDING_TO_43S
REACTOME_ASSOCIATION_OF_TRIC_CCT_WITH_TARGET_PROTEINS_DURING_BIOSYNTHESIS
REACTOME_PROTEIN_FOLDING
REACTOME_PREFOLDIN_MEDIATED_TRANSFER_OF_SUBSTRATE_TO_CCT_TRIC
REACTOME_DOPAMINE_NEUROTRANSMITTER_RELEASE_CYCLE
TRANSMISSION_OF_NERVE_IMPULSE
REACTOME_NEUROTRANSMITTER_RELEASE_CYCLE
SYNAPTIC_TRANSMISSION
ACTIVE_TRANSMEMBRANE_TRANSPORTER_ACTIVITY
COATED_VESICLE
CLATHRIN_COATED_VESICLE
REACTOME_APOPTOSIS_INDUCED_DNA_FRAGMENTATION
SECOND_MESSENGER_MEDIATED_SIGNALING
NUCLEASE_ACTIVITY
CHROMATIN
Enriched in Non-responders
Enriched in Control

Supplemental Figure S5. GSEA enrichment map for significantly affected pathways (p-value < 0.05, false discovery rate < 25%) in **(A)** responders relative to controls, and **(B)** non-responders relative to control using the Reactome and Gene Ontology datasets. Red dots indicate positively enriched pathways; blue indicates negatively enriched pathways. Relative size maps to relative magnitude of Normalized enrichment score. Edge size corresponds to number of proteins that overlap between gene sets.

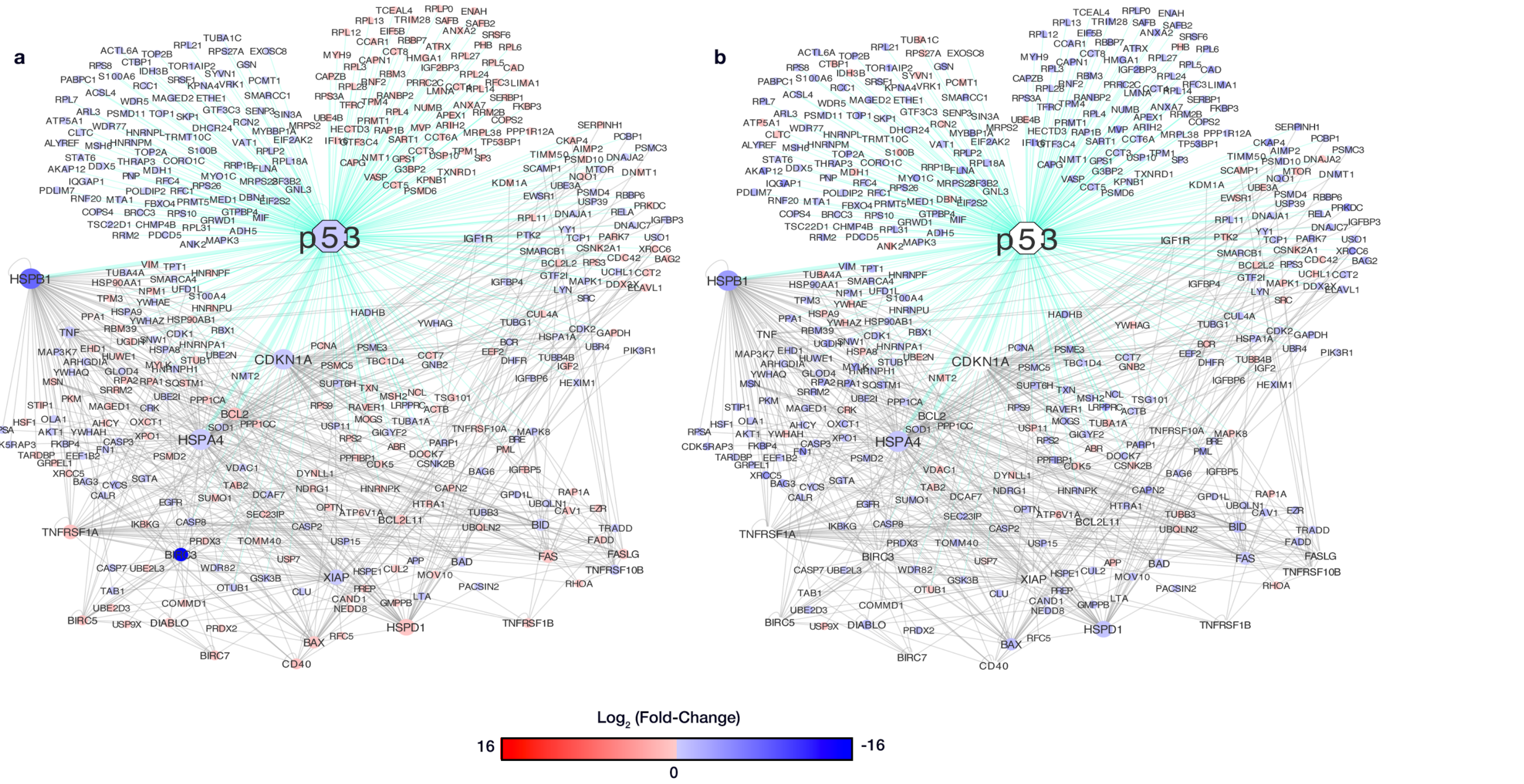
a
b
p53
HSPB1
CDKN1A
HSPA4
BCL2
TNFRSF1A
BIRC3
XIAP
HSPD1
BAX
Log2 (Fold-Change)
16
0
-16

Supplemental Figure S6. Gene neighborhood network (via BIOGRID and g:profiler) using proteins from apoptosis antibody array. Coloring indicated average protein fold-change for **(a)** non-responders, and **(b)** responders relative to controls using proteomics and western array data. Network has been pruned for visualization for any undetected/unmeasured protein and any node of degree 1 that was not directly connected to p53. Overall this network in non-responders is mixed in regulation, while the responder network is primarily down-regulated relative to controls.

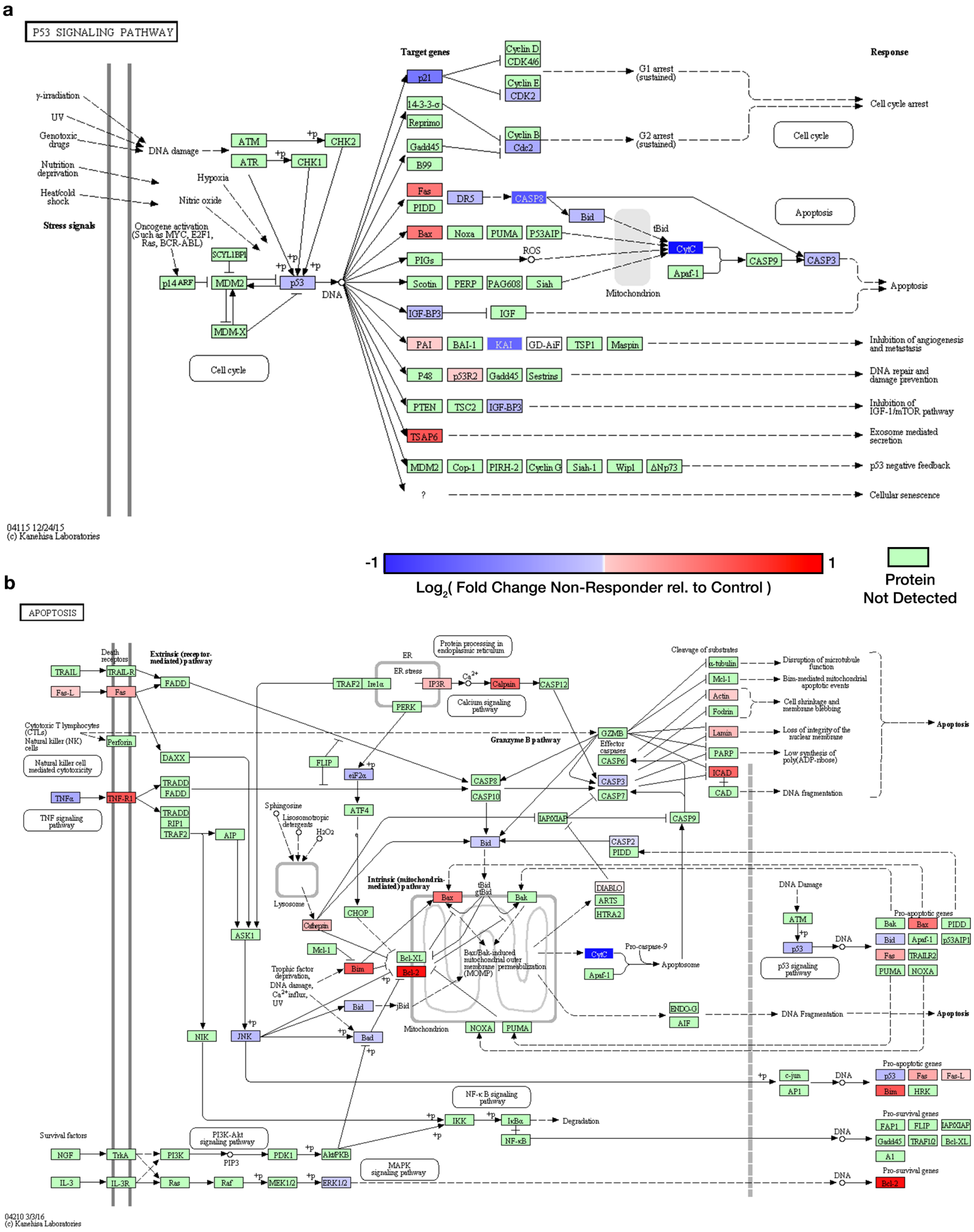

a
P53 SIGNALING PATHWAY
γ-irradiation
UV
Genotoxic drugs
Nutrition deprivation
Heat/cold shock
Stress signals
DNA damage
ATM
ATR
CHK2
CHK1
Hypoxia
Nitric oxide
Oncogene activation (Such as MYC, E2F1, Ras, BCR-ABL)
SCYL1BP1
p14ARF
MDM2
p53
MDM-X
DNA
Cell cycle
Target genes
p21
14-3-3-σ
Reprimo
Gadd45
B99
Cyclin D
CDK4/6
Cyclin E
CDK2
Cyclin B
Cdc2
G1 arrest (sustained)
G2 arrest (sustained)
Response
Cell cycle arrest
Cell cycle
Fas
PIDD
DR5
CASP8
Bid
tBid
Bax
Noxa
PUMA
P53AIP
PIGs
ROS
CytC
Apaf-1
CASP9
CASP3
Scotin
PERP
PAG608
Siah
Mitochondrion
Apoptosis
IGF-BP3
IGF
PAI
BAI-1
KAI
GD-AiF
TSP1
Maspin
Inhibition of angiogenesis and metastasis
P48
p53R2
Gadd45
Sestrins
DNA repair and damage prevention
PTEN
TSC2
IGF-BP3
Inhibition of IGF-1/mTOR pathway
TSAP6
Exosome mediated secretion
MDM2
Cop-1
PIRH-2
Cyclin G
Siah-1
Wip1
ΔNp73
p53 negative feedback
?
Cellular senescence
04115 12/24/15
(c) Kanehisa Laboratories
-1
1
Log2(Fold Change Non-Responder rel. to Control)
Protein Not Detected
b
APOPTOSIS
TRAIL
TRAIL-R
Fas-L
Fas
Death receptors
Extrinsic (receptor-mediated) pathway
FADD
Cytotoxic T lymphocytes (CTLs)
Natural killer (NK) cells
Perforin
Natural killer cell mediated cytotoxicity
DAXX
TNFα
TNF-R1
TRADD
FADD
RIP1
TRAF2
TNF signaling pathway
AIP
ER
ER stress
TRAF2
Ire1α
PERK
IP3R
Ca2+
Calpain
CASP12
Protein processing in endoplasmic reticulum
Calcium signaling pathway
FLIP
eiF2α
ATF4
CHOP
CASP8
CASP10
Granzyme B pathway
GZMB
Effector caspases
CASP6
CASP3
CASP7
IAP/XIAP
CASP9
Bid
CASP2
PIDD
Sphingosine
Lisosomotropic detergents
H2O2
Lysosome
Cathepsin
ASK1
Intrinsic (mitochondria-mediated) pathway
tBid
gBid
Bax
Bak
DIABLO
ARTS
HTRA2
Mcl-1
Bim
Bcl-XL
Bcl-2
Trophic factor deprivation, DNA damage, Ca2+ influx, UV
Bax/Bak-induced mitochondrial outer membrane permeabilization (MOMP)
CytC
Pro-caspase-9
Apaf-1
Apoptosome
Bid
jBid
Bad
JNK
NIK
Mitochondrion
NOXA
PUMA
ENDO-G
AIF
DNA Fragmentation
Apoptosis
Cleavage of substrates
α-tubulin
Mcl-1
Actin
Fodrin
Lamin
PARP
ICAD
CAD
Disruption of microtubule function
Bim-mediated mitochondrial apoptotic events
Cell shrinkage and membrane blebbing
Loss of integrity of the nuclear membrane
Low synthesis of poly(ADP-ribose)
DNA fragmentation
Apoptosis
DNA Damage
ATM
p53
DNA
p53 signaling pathway
Pro-apoptotic genes
Bak
Bax
PIDD
Bid
Apaf-1
p53AIP1
Fas
TRAILR2
PUMA
NOXA
c-jun
AP1
DNA
Pro-apoptotic genes
p53
Fas
Fas-L
Bim
HRK
NF-κB signaling pathway
IKK
IκBα
NF-κB
Degradation
Pro-survival genes
FAP1
FLIP
IAP/XIAP
Gadd45
TRAF1/2
Bcl-XL
A1
Survival factors
NGF
TrkA
PI3K
PIP3
PDK1
Akt/PKB
PI3K-Akt signaling pathway
IL-3
IL-3R
Ras
Raf
MEK1/2
ERK1/2
MAPK signaling pathway
Pro-survival genes
Bcl-2
04210 3/3/16
(c) Kanehisa Laboratories

Supplemental Figure S7. KEGG **(a)** P53 Signaling (HAS:04115) and **(b)** Apoptosis (HSA:04210) pathways, colored using average non-responder fold-change relative to controls, using proteomics and western array data. No clear pro- or anti-apoptotic signal is apparent from these mappings i.e., both relatively positive and negative expression of proteins occurs for both pro- and anti-apoptotic responses.

Supplemental Table S1 Primers used for cloning and FISH probe

Primer Name	Primer sequence (5′ to 3 ′)
PflE-F	ATATACATGTAAACGGCCTCTCTTATTTC
PflE-R	ATATAAGCTTCCACTTTACGGATAAACATACC
p53-F	ATATGGATCCATGGAGGAGCCGCAGTCAGATCC
p53-R	ATATGAATTCTCAGTCTGAGTCAGGCCCTTCTGTC
Azurin-F	ATATGGATCCACGCCGGGCAGATCCCGCTC
Azurin-R	ATATGAATTCTCACTTCAGGGTCAGGGTGCCCTTCATC
23S rDNA FISH probe with 5′Texas Red	AATCACTTCACCTACGTG

Supplemental Table S2. Mass spectrometry results for identification of Azurin expression (3 biological replicates). Listed are the results detected as AZUR_PSEAE (accession: P00282, species: Pseudomonas aeruginosa). Azurin-negative controls did not have a detectable gel band for mass spectrometry analysis.

Sample ID	Sample Group	# Unique Peptides	Intensity
5	Azurin+	5	1.20E+08
6	Azurin+	5	2.10E+08
7	Azurin+	2	1.40E+07

Supplemental Table S3. Proteomic individual protein snapshot. Shown are the 20 largest by Log2 Fold Change (Log2 FC) magnitude, up- and down- fold changes between each condition pair. Values below the significance threshold are highlighted in red. Please see **Supplemental Information** for complete dataset.

**Non-responders relative to Controls**				**Responders relative to Controls**				**Responders relative to Non-responders**			
**UP Fold**				**UP Fold**				**UP Fold**			
Gene	Accession	Log2 FC	p-value	Gene	Accession	Log2 FC	p-value	Gene	Accession	Log2 FC	p-value
ALDH1A3	P47895	1.387	0.000606033	Ppp1r9a	O35867	2.882	9.60E-16	Pclo	Q9JKS6	4.549	2.80E-11
HSPB6	O14558	1.35	0.000363856	NEGR1	Q7Z3B1	2.799	8.23E-13	Bsn	G3V984	4.36	2.98E-24
ETFA	H0YM12	1.195	0.00080317	Slc6a17	P31662	2.758	2.17E-12	Bsn	O88778	4.184	4.34E-27
SDCBP	G5EA09	1.143	2.26E-06	Madd	O08873	2.682	7.51E-13	Gria2	F1LNE4	4.139	8.54E-08
Itgam	G3V8L7	1.13	0.001078139	Rap1gap	F1LV89	2.668	2.47E-09	Tnr	A0A096MJE6	3.971	3.38E-14
VPS51	Q9UID3	1.091	0.049194741	Caskin1	D3ZE17	2.633	1.20E-15	SYNGAP1	Q96PV0	3.964	8.35E-10
Ufc1	Q6BBI8	1.023	0.030357066	Ppp1r1b	Q6J4I0	2.603	1.29E-13	Dmxl2	F1M164	3.932	4.19E-08
Arfgef2	Q7TSU1	1.002	0.034141206	Negr1	Q9Z0J8	2.596	3.24E-13	Plcb1	P10687	3.857	3.91E-13
CCDC124	Q96CT7	0.999	0.009064815	Ppfia3	F1LSE6	2.575	1.19E-13	Palm	Q920Q0	3.739	2.22E-14
Lsm2	Q6MG66	0.996	0.010576147	PTPRD	P23468	2.496	6.11E-08	Pde2a	F8WFW5	3.66	2.62E-10
CTSA	X6R5C5	0.982	0.002856246	CALB1	P05937	2.493	2.62E-13	Srcin1	Q9QXY2	3.543	6.77E-20
H1FX	Q92522	0.972	0.05456802	Cep170b	D4A1G8	2.488	6.05E-08	Ank2	F1LM42	3.529	5.92E-36
UQCRH	P07919	0.966	0.022953964	Agap2	Q8CGU4	2.416	7.21E-12	Oxr1	Q4V8B0	3.505	3.76E-11
RRP8	O43159	0.959	0.040943669	Myef2	D4AEI5	2.373	6.39E-11	DMXL2	Q8TDJ6-3	3.453	3.49E-12
TUBGCP2	Q9BSJ2-4	0.95	0.02492262	Scn2a	P04775	2.349	2.21E-14	Camkv	F1LR80	3.45	9.14E-13
SRP19	P09132	0.947	0.03322118	Acsl6	A0A0A6YYM0	2.334	2.37E-16	SYT1	P21579	3.445	1.50E-35
Emr1	Q5Y4N8	0.928	0.065066637	Akap12	Q5QD51	2.327	1.12E-14	Synpo	Q9Z327-2	3.432	2.44E-08
HNMT	P50135	0.927	0.054959028	Pclo	Q9JKS6	2.324	1.26E-38	Camkv	Q63092	3.426	8.67E-12
Vps53	D3ZPE5	0.922	0.051045151	Dpp10	Q6Q629	2.322	5.79E-11	Add2	F8WFS9	3.424	9.44E-11
Cstf2t	M0R6B9	0.911	0.046867016	Prkce	P09216	2.317	1.20E-10	Impact	Q5GFD9	3.413	3.74E-08

Non-responders relative to Controls

DOWN Fold

Gene	Accession	Log2 FC	p-value
Bsn	G3V984	-2.622	1.44E-06
Bsn	O88778	-2.531	6.74E-06
Tnr	A0A096MJE6	-2.218	0.0004623
Plcb1	P10687	-2.123	8.15E-05
Pgrmc1	P70580	-1.97	0.002310608
NCDN	Q9UBB6-3	-1.956	9.16E-05
Hpcal4	P35332	-1.94	0.003098431
Scin	E9PU64	-1.905	0.000154763
Pclo	Q9JKS6	-1.889	0.003141926
Dmxl2	F1M164	-1.851	0.003603893
KRT2	P35908	-1.816	0.000899208
Synpo	Q9Z327	-1.806	0.005110261
Ncdn	O35095	-1.765	0.000144521
Synpo	Q9Z327-2	-1.74	0.008823966
Tmod2	P70566	-1.728	0.003512689
Homer1	Q9Z214	-1.718	0.012052618
Gria2	F1LNE4	-1.714	0.012457946
Camkv	Q63092	-1.704	0.001715614
Add2	F8WFS9	-1.696	0.00342469
L1cam	D3ZPC4	-1.668	0.007714873

Responders relative to Controls

DOWN Fold

Gene	Accession	Log2 FC	p-value
COL6A3	P12111	-4.848	3.75E-28
A1i3	P14046	-4.794	1.85E-14
PRKDC	P78527	-4.561	2.65E-13
Mug1	Q03626	-4.544	1.86E-15
TNC	P24821	-4.449	1.72E-13
ANXA1	P04083	-4.441	9.92E-15
NES	P48681	-4.425	4.68E-12
IQGAP1	P46940	-4.416	4.48E-11
FN1	P02751-15	-4.352	8.74E-11
FLNC	Q14315	-4.272	2.27E-16
AHNAK2	Q8IVF2	-4.256	2.39E-12
MVP	Q14764	-4.115	1.74E-19
TGFBI	Q15582	-4.056	1.83E-11
COL6A1	P12109	-4.035	8.09E-11
Iqgap1	G3V7Q7	-4.019	1.63E-11
KTN1	Q86UP2	-3.971	1.85E-11
A1m	Q63041	-3.955	1.22E-12
Hpx	P20059	-3.933	4.86E-10
CAST	P20810-6	-3.928	8.33E-10
COL6A2	P12110	-3.919	1.38E-10

Responders relative to Non-responders

DOWN Fold

Gene	Accession	Log2 FC	p-value
COL6A3	P12111	-5.025	4.84E-26
PRKDC	P78527	-4.763	3.28E-13
ANXA1	P04083	-4.596	3.56E-13
TGFBI	Q15582	-4.528	7.52E-14
A1i3	P14046	-4.516	8.03E-13
Mug1	Q03626	-4.509	1.69E-11
NES	P48681	-4.473	7.03E-12
FBN1	P35555	-4.452	8.32E-14
AHNAK2	Q8IVF2	-4.451	3.77E-13
IQGAP1	P46940	-4.361	3.20E-13
COL6A1	P12109	-4.313	2.93E-11
TNC	P24821	-4.26	3.84E-13
LGALS1	P09382	-4.26	6.71E-56
MVP	Q14764	-4.206	2.87E-19
FN1	P02751-15	-4.196	4.12E-10
CAST	P20810-6	-4.19	1.24E-11
KTN1	Q86UP2	-4.119	1.03E-11
FAM129B	Q96TA1	-4.086	1.96E-12
PLIN3	O60664	-4.028	1.16E-10
CKAP4	Q07065	-4.002	1.02E-10

Supplemental Table S4. Gene Set Enrichment Analysis (GSEA) snapshot. Shown are the 5 largest positively (red) and negatively (blue) enriched pathways for each condition pair, selected by Normalized Enrichment Score (NES) magnitude. ES: Enrichment score; NOM p-val: nominal p-value given by GSEA; FDR: False Discovery Rate; FWER: Family-wise error rate.

	NAME	SIZE	ES	NES	NOM p-val	FDR q-val	FWER p-val
**Non-responders**	PROTEIN FOLDING	17	0.6480	2.4384	0.0000	0.0186	0.0090
relative to	ASSOCIATION OF TRIC CCT WITH TARGET PROTEINS DURING BIOSYNTHESIS	10	0.7606	2.3412	0.0000	0.0170	0.0170
**Controls**	FORMATION OF THE TERNARY COMPLEX AND SUBSEQUENTLY THE 43S COMPLEX	11	0.6838	2.1303	0.0000	0.0788	0.1100
	PEPTIDE CHAIN ELONGATION	33	0.4547	2.1004	0.0000	0.0797	0.1470
	STRUCTURAL CONSTITUENT OF RIBOSOME	27	0.4858	2.0839	0.0000	0.0735	0.1690
	TRANSMISSION OF NERVE IMPULSE	34	-0.6387	-1.6677	0.0000	0.1472	0.6770
	CLATHRIN COATED VESICLE	9	-0.8026	-1.6971	0.0000	0.0985	0.4820
	NEUROTRANSMITTER RELEASE CYCLE	16	-0.7191	-1.7001	0.0021	0.1114	0.4680
	SECOND MESSENGER MEDIATED SIGNALING	14	-0.7356	-1.7007	0.0011	0.1365	0.4610
	SYNAPTIC TRANSMISSION	30	-0.6641	-1.7201	0.0000	0.1147	0.3260
**Responders**	TCA CYCLE AND RESPIRATORY ELECTRON TRANSPORT	74	0.5741	2.3851	0.0000	0.0000	0.0000
relative to	TRANSMISSION ACROSS CHEMICAL SYNAPSES	60	0.5787	2.2875	0.0000	0.0000	0.0000
**Controls**	NEURONAL SYSTEM	66	0.5587	2.2337	0.0000	0.0000	0.0000
	RESPIRATORY ELECTRON TRANSPORT ATP SYNTHESIS BY CHEMIOSMOTIC COUPLING AND HEAT PRODUCTION BY UNCOUPLING PROTEINS	49	0.5782	2.2141	0.0000	0.0000	0.0000
	MITOCHONDRION	114	0.4766	2.1351	0.0000	0.0003	0.0020
	NONSENSE MEDIATED DECAY ENHANCED BY THE EXON JUNCTION COMPLEX	35	-0.7143	-2.8738	0.0000	0.0000	0.0000
	PEPTIDE CHAIN ELONGATION	33	-0.7578	-2.8885	0.0000	0.0000	0.0000
	INFLUENZA VIRAL RNA TRANSCRIPTION AND REPLICATION	34	-0.7467	-2.8986	0.0000	0.0000	0.0000
	3 UTR MEDIATED TRANSLATIONAL REGULATION	36	-0.7423	-2.9374	0.0000	0.0000	0.0000

	NAME	SIZE	ES	NES	NOM p-val	FDR q-val	FWER p-val
**Responders**	NEUROTRANSMITTER RELEASE CYCLE	17	0.7612	2.0635	0.0000	0.0031	0.0040
relative to	ACTIVE TRANSMEMBRANE TRANSPORTER ACTIVITY	30	0.6512	1.9963	0.0000	0.0062	0.0150
**Non-responders**	NEURONAL SYSTEM	69	0.5640	1.9732	0.0000	0.0065	0.0230
	SYNAPTIC TRANSMISSION	31	0.6445	1.9725	0.0000	0.0049	0.0230
	TCA CYCLE AND RESPIRATORY ELECTRON TRANSPORT	77	0.5515	1.9322	0.0000	0.0071	0.0440
	REACTOME 3 UTR MEDIATED TRANSLATIONAL REGULATION	36	-0.7416	-3.0801	0.0000	0.0000	0.0000
	REACTOME INFLUENZA VIRAL RNA TRANSCRIPTION AND REPLICATION	34	-0.7420	-3.0824	0.0000	0.0000	0.0000
	REACTOME SRP DEPENDENT COTRANSLATIONAL PROTEIN TARGETING TO MEMBRANE	39	-0.7320	-3.0827	0.0000	0.0000	0.0000
	REACTOME INFLUENZA LIFE CYCLE	45	-0.7101	-3.1372	0.0000	0.0000	0.0000
	REACTOME TRANSLATION	52	-0.7248	-3.2511	0.0000	0.0000	0.0000
